# Composite Hydrogel Using Methacrylated Silk Fibroin and Mercaptolated Hyaluronic Acid with Encapsulating Zinc-Quercetin Nanozyme

**DOI:** 10.3390/gels12080665

**Published:** 2026-07-24

**Authors:** Lei Nie, Xinran Li, Ruqiang Gong, Han Zhang, Guohua Jiang

**Affiliations:** 1 College of Life Sciences, Xinyang Normal University, Xinyang 464000, China; 2 School of Materials Science and Engineering, Zhejiang Sci-Tech University, Hangzhou 310018, China; 3 International Scientific and Technological Cooperation Base of Intelligent Biomaterials and Functional Fibers, Hangzhou 310018, China; 4 Zhejiang Provincial Key Laboratory of Silk and Silk Protein Materials, Hangzhou 310018, China

**Keywords:** silk fibroin, hyaluronic acid, nanozyme, hydrogel

## Abstract

Given the urgent need to regulate oxidative stress microenvironments in chronic wound healing, hydrogel dressings that simultaneously integrate antioxidant, antibacterial, mechanically adaptive, and biocompatible properties are highly desirable. In this study, a natural polymer-based composite hydrogel dressing loaded with zinc-quercetin nanozyme (Zn-Q) was designed. The gel skeleton was constructed via a dual network of photocrosslinked methacrylated silk fibroin (SilMA) and mercaptolated hyaluronic acid (HA-SH) via thiol-ene click chemistry, with the catalase (CAT)-like Zn-Q nanozyme encapsulated in situ within the network, thereby achieving synergy between chemical crosslinking and dynamic metal-polyphenol coordination. Systematic characterization revealed that Zn-Q nanozyme adopted a stable octahedral coordination configuration, and its continuous porous structure exposed abundant catalytically active sites. The composite hydrogels exhibited a highly interconnected, three-dimensional (3D) porous morphology, with swelling ratios that increased significantly with Zn-Q nanozyme content (up to around 1082%). Rheological and mechanical tests demonstrated that although incorporating the nanozyme reduced the storage modulus, the reversible physical crosslinks formed via hydrogen bonding and coordination interactions endowed the material with excellent tensile toughness and energy-dissipation capacity, exhibiting typical Mullins softening behavior. Functional evaluation showed that Zn-Q nanozyme conferred superior free radical scavenging capability to the hydrogels and exerted dose-dependent inhibition against both *Staphylococcus aureus* and *Escherichia coli*. Furthermore, the hydrogels exhibited favorable adhesion to various wet organs and heterogeneous material surfaces, with hemolysis rates below 5% and cell viability exceeding 100% after 3 days of culturing with fibroblasts, confirming their excellent hemocompatibility and cytocompatibility. This study provides an experimental basis for developing a new type of wound repair materials that integrate antioxidant, anti-infective, and mechanically adaptive properties, holding significant application potential in oxidative stress-related tissue repair fields.

## 1. Introduction

Impaired wound healing, particularly in chronic conditions, remains a major clinical challenge due to persistent inflammation, excessive reactive oxygen species (ROS), and recurrent bacterial infections [1,2]. The accumulation of ROS disrupts the homeostasis of the tissue microenvironment, inhibits cell proliferation and migration, and delays re-epithelialization [3,4]. Consequently, wound dressings that can effectively scavenge ROS while providing antibacterial protection and suitable mechanical support are highly desirable. Hydrogels, with their three-dimensional hydrophilic networks that mimic the extracellular matrix (ECM), have emerged as promising candidates for wound management because they maintain a moist wound environment, facilitate oxygen and nutrient exchange, and can be engineered to deliver bioactive agents [5,6]. Silk fibroin (SF), derived from *Bombyx mori* silkworm cocoons, is a natural protein renowned for its excellent biocompatibility, tunable mechanical properties, and low immunogenicity [7,8,9]. To render SF photocrosslinkable, methacryloyl groups are introduced via reaction with glycidyl methacrylate (GMA), yielding methacrylated silk fibroin (SilMA) [10]. SilMA can form stable hydrogels under UV irradiation in the presence of a photoinitiator, offering controlled gelation kinetics and mechanical tunability. However, pure SilMA hydrogels often exhibit brittleness and insufficient bioactivity for complex wound-healing processes [11,12]. Hyaluronic acid (HA), a non-sulfated glycosaminoglycan abundant in the ECM, plays a critical role in wound repair by promoting cell migration, angiogenesis, and tissue hydration [13,14]. Chemical modification of HA with thiol groups (–SH) via amidation with cysteamine yields mercaptolated hyaluronic acid (HA-SH) [15], which can participate in thiol-ene click reactions, forming additional crosslinks and improving network flexibility and biofunctionality [16].

The integration of SilMA and HA-SH into a dual-network matrix strategically addresses the multifaceted requirements of an ideal wound dressing, encompassing mechanical robustness, biological functionality, and crosslinking versatility. While the photocrosslinked SilMA network provides high modulus and structural integrity, its dense methacryloyl crosslinks induce brittleness [17]. In contrast, HA-SH imparts flexible glycan chains that introduce viscoelasticity and extensibility to the network [18,19]. The interpenetrating or semi-interpenetrating architecture of the SilMA/HA-SH hydrogel allows efficient stress transfer between the stiff SilMA backbone and the compliant HA-SH segments, preventing catastrophic failure under load [20]. These flexible chains act as “molecular springs” that dissipate energy through uncoiling and reorientation, thereby transforming the brittle SilMA network into a tough composite that accommodates tissue movements and external compression [21]. In addition, SilMA undergoes rapid radical polymerization upon UV exposure, enabling fast in situ gelation and immediate shape fixation, which is considered an advantage for minimally invasive applications [22]. Meanwhile, the thiol groups on HA-SH can undergo thiol-Michael addition with the unreacted methacryloyl groups of SilMA, forming covalent thioether bonds under mild physiological conditions without additional catalysts or harsh reagents [18]. This orthogonal dual-crosslinking strategy, including photocrosslinking and click chemistry, allows decoupled control over network topology: the primary SilMA framework provides the overall elastic modulus, while the secondary thiol-ene crosslinks fine-tune the mesh size, crosslinking density, and network homogeneity. Moreover, the reversible nature of thiol-disulfide exchange, such as that driven by residual thiols or oxidation, introduces dynamic covalent bonds that contribute to stress relaxation and energy dissipation via a “sacrificial bond” mechanism [19,23].

The chemical orthogonality of the two crosslinking reactions of SilMA and HA-SH offers remarkable flexibility for incorporating additional functional moieties, such as the nanozyme. The photocrosslinking of methacryloyl groups and the thiol-ene click reaction proceed independently, without interference, allowing the homogeneous encapsulation of nanoparticles without compromising gelation kinetics or network integrity. The abundant carboxyl, hydroxyl, and amide groups on both polymers further facilitate multiple noncovalent interactions (hydrogen bonding, electrostatic interactions) with the phenolic hydroxyl and coordinated carbonyl groups of the specific nanozyme, anchoring the nanozyme within the network and modulating its release profile [24]. Furthermore, to address the oxidative stress and infection challenges, we incorporated a zinc-quercetin nanozyme (Zn-Q) into the hydrogel matrix. Quercetin, a natural flavonoid, possesses potent antioxidant activity due to its ortho-diphenolic hydroxyl groups, which can directly scavenge free radicals and chelate metal ions [25,26]. When coordinated with Zn^2+^ ions, quercetin forms stable metal-phenolic networks (Zn-Q) that exhibit catalase (CAT)-like activity, efficiently decomposing H_2_O_2_ into water and oxygen [27]. The Zn-Q nanozyme adopts an octahedral coordination geometry, preserving the conjugated aromatic skeleton of quercetin and providing essential electron-transfer pathways for enzyme-mimetic catalysis [28]. Moreover, the released Zn^2+^ ions and quercetin molecules impart antibacterial effects by disrupting bacterial cell membranes and inducing oxidative stress in pathogens [29,30]. Recently, Hadisi et al. reported a hyaluronic acid-based silk fibroin core–shell electrospun dressing loaded with zinc oxide for burn wound management, demonstrating that incorporating zinc-based antibacterial agents into SF/HA systems can effectively enhance antibacterial activity and promote wound healing in a dose-dependent manner. However, electrospun nanofibrous dressings differ fundamentally from hydrogel systems in water content, mechanical properties, and drug-release kinetics [31]. Our SilMA/HA-SH/Zn-Q hydrogel platform offers advantages including a moist wound environment, tunable viscoelasticity, and three-dimensional cell penetration, which are particularly beneficial for chronic wound healing.

**Scheme 1:**
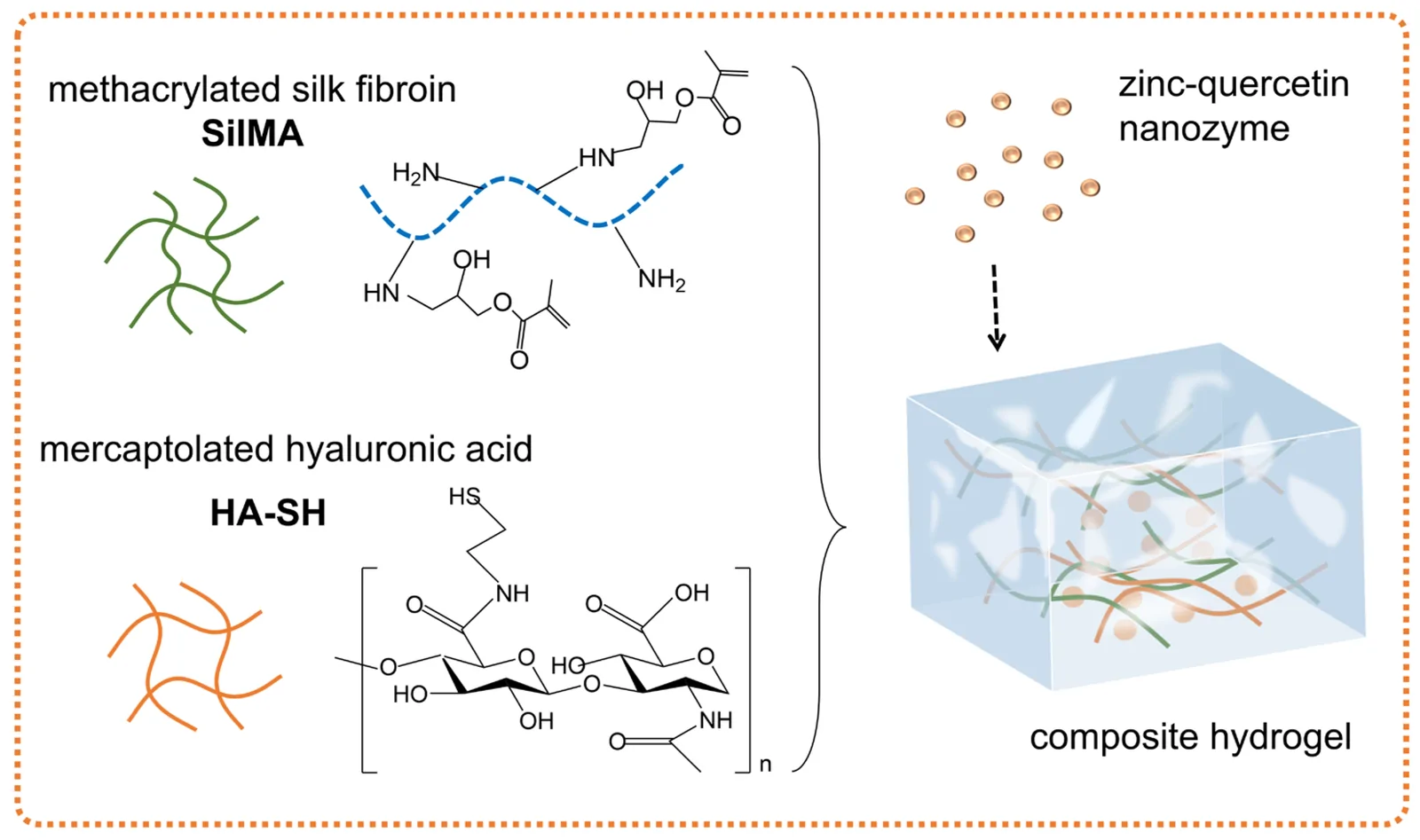
Schematic diagram showing the preparation of multifunctional polysaccharide-based hydrogels using methacrylated silk fibroin, mercaptolated hyaluronic acid, and a zinc-quercetin nanozyme.

The overall design rationale of this study is to synergistically integrate the structural advantages of the SilMA/HA-SH dual network with the multifunctional bioactivity of Zn-Q nanozyme (Scheme 1). The photocrosslinked SilMA provides the primary elastic framework, and HA-SH introduces additional thiol-ene crosslinks, biological signaling, and enhanced hydrophilicity. The Zn-Q nanozyme serves as an antioxidant via direct radical scavenging and CAT-mimetic activity, an antibacterial agent through Zn^2+^ release and membrane disruption, and a dynamic physical crosslinker that modulates mechanical properties through reversible noncovalent interactions. This synergistic combination is expected to yield hydrogels with tunable swelling, robust adhesion, excellent cytocompatibility, and enhanced wound-healing potential. The physicochemical properties of the fabricated hydrogels, including microstructure, swelling behavior, rheological properties, and mechanical performance (tensile, compressive, and cyclic loading), were systematically evaluated. Furthermore, the antioxidant capacity, antibacterial activity against *E. coli* and *S. aureus*, tissue adhesion, hemocompatibility, and cytocompatibility were assessed.

## 2. Results and Discussion

### 2.1. Synthesis of SilMA, HA-SH and Zn-Q Nanozyme

**Figure 1:**
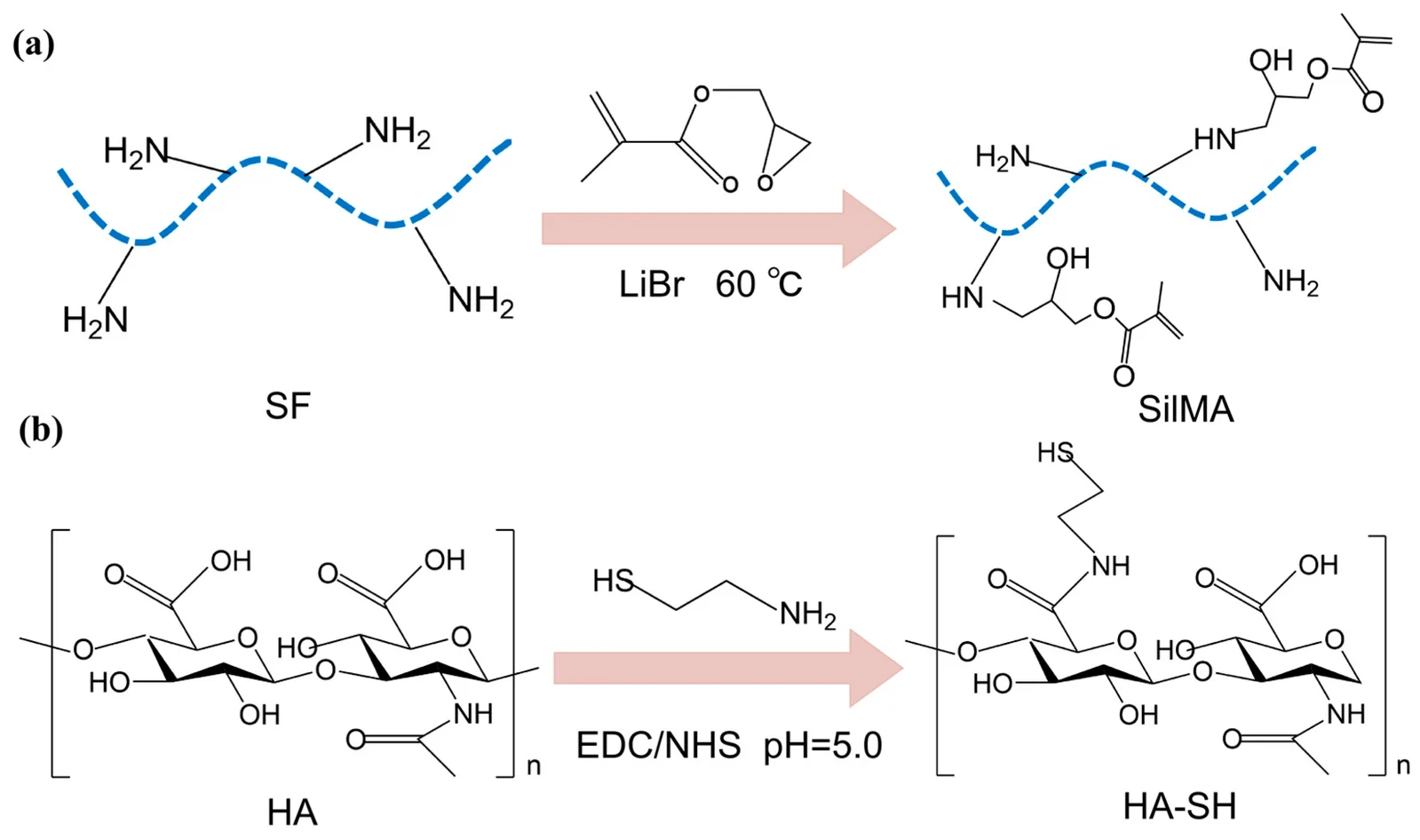
Schematic illustration of the synthesis routes of (**a**) SilMA and (**b**) HA-SH.

This study achieved the functional modification of silk fibroin and hyaluronic acid. As shown in Figure 1a, the abundant amino groups on the silk fibroin (SF) molecular chains underwent a ring-opening reaction with the epoxy groups of glycidyl methacrylate (GMA), introducing methacryloyl groups into the side chains of SF to produce methacrylated silk fibroin (SilMA). Meanwhile, under the action of the EDC/NHS coupling system, the carboxyl groups on the hyaluronic acid (HA) chains reacted with the amino groups of cysteamine via amidation (Figure 1b), successfully introducing thiol groups (-SH) into HA to obtain mercaptolated hyaluronic acid (HA-SH).

**Figure 2:**
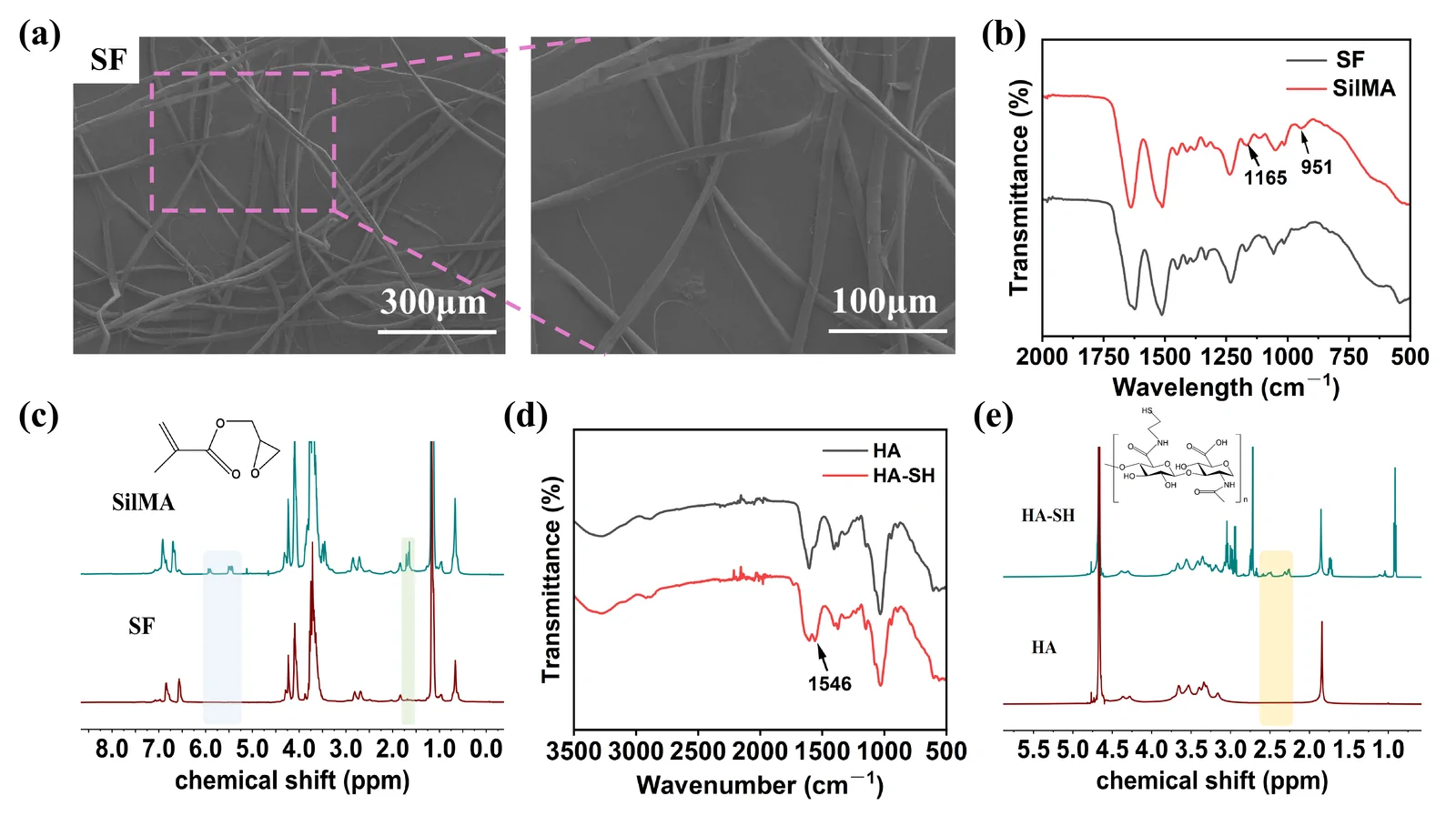
(**a**) SEM images of SF. (**b**) FT-IR spectra and (**c**) ^1^H NMR spectra of SF and SilMA. (**d**) FT-IR spectra and (**e**) ^1^H NMR spectra of HA and HA-SH.

In Figure 2a, the degummed SF exhibited a typical fibrous morphology. The chemical structures of the synthesized products were characterized using FT-IR and ^1^H NMR. As shown in Figure 2b, compared with the raw SF, SilMA displayed new characteristic absorption peaks at 1165 cm^−1^ and 951 cm^−1^, which were assigned to the C-O-C ether bond and the terminal C=C bond of the methacryloyl group, indicating successful grafting of GMA onto the SF chains [32]. The ^1^H NMR spectrum of SilMA (Figure 2c) exhibited two new proton signals at chemical shifts of 5.5–6.0 ppm (blue shaded area) and 1.8–2.0 ppm (green shaded area), corresponding to the resonance absorptions of the terminal double bond (=CH_2_) and the methyl group (-CH_3_) of the methacryloyl moiety, respectively, which are consistent with the reported NMR features of SilMA [33]. The degree of substitution (DS) of MA groups in SilMA was 54%, as determined by integration of the ^1^H NMR spectrum. As shown in Figure 2d, compared with HA, HA-SH exhibited a significantly enhanced absorption peak at 1546 cm^−1^, which corresponds to the amide II band (coupled N-H bending and C-N stretching) of the amide bond [34]. The S-H stretching vibration (typically 2550 cm^−1^) was not clearly observed due to its inherently weak infrared absorption, necessitating confirmation by ^1^H NMR spectroscopy (Figure 2e). HA-SH exhibited new proton signals at 2.6–2.8 ppm (yellow-shaded area), attributed to the methylene protons (-CH2-) adjacent to the thiol group in the cysteamine moiety, confirming the successful thiolation of HA, with a degree of substitution of 39.8% [35].

**Figure 3:**
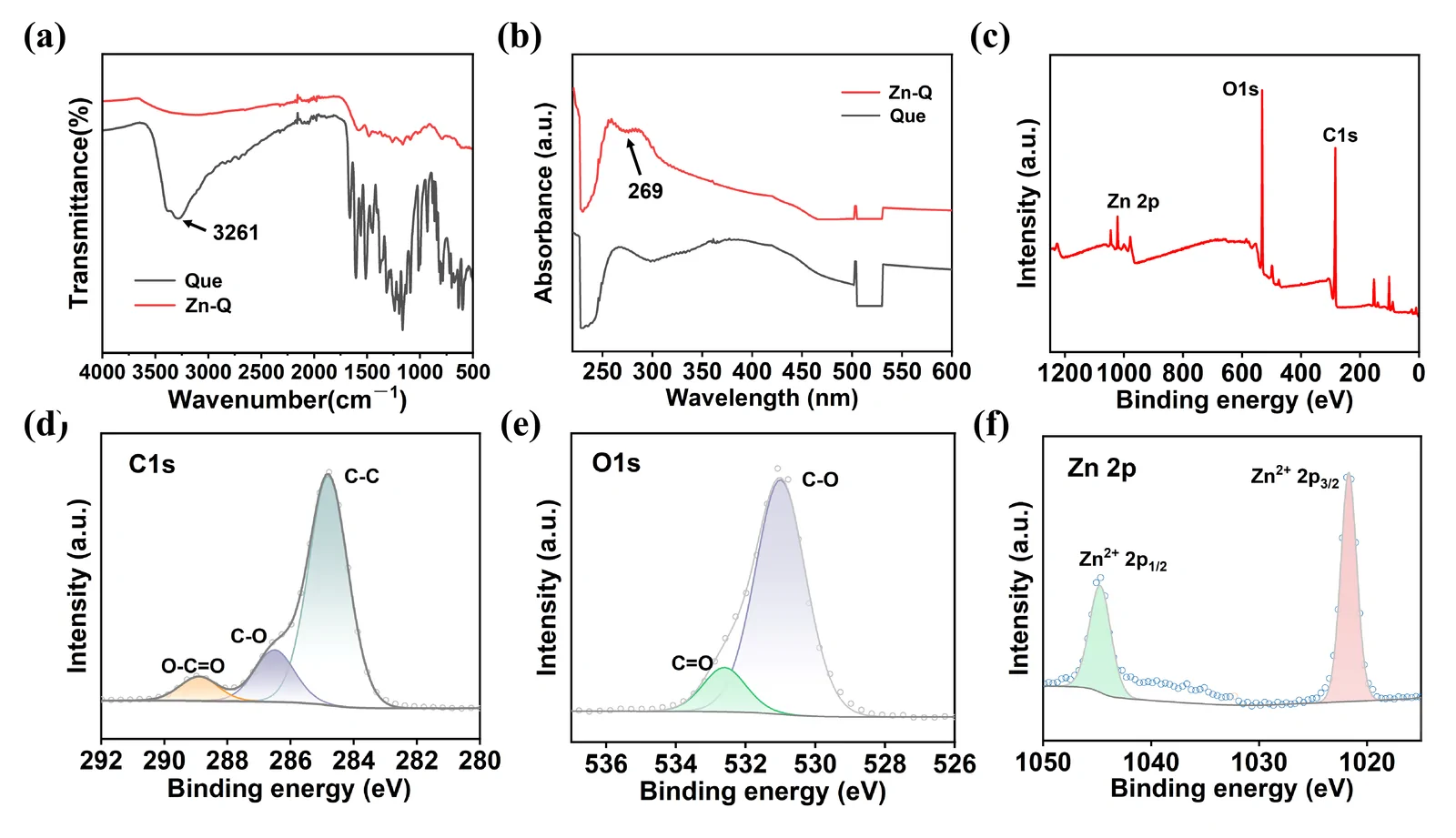
(**a**) FT-IR spectra and (**b**) UV–Vis spectra of Que and Zn-Q nanozyme. (**c**) XPS survey spectrum of the Zn-Q nanozyme. High-resolution XPS spectra of (**d**) C1s, (**e**) O1s, and (**f**) Zn2p of the Zn-Q nanozyme.

To confirm the coordination between zinc ions and quercetin (Que), the Zn-Q nanozyme was characterized by spectroscopic methods. The FT-IR spectrum (Figure 3a) showed that, compared with quercetin, the Zn-Q nanozyme exhibited a significantly weakened hydroxyl (-OH) stretching vibration peak at 3261 cm^−1^, as well as distinct changes and shifts in the peak shape within the 1600–1400 cm^−1^ region, which correspond to the benzene ring skeletal vibrations and C=O stretching vibrations [36]. Previous studies have confirmed that Zn^2+^ can form stable metal-ligand coordination bonds with the carbonyl oxygen atom (C=O) and the C-OH group (ring II) of the quercetin ligand [37]. The UV–Vis absorption spectrum (Figure 3b) further supported the formation of the coordination structure. Quercetin exhibited two characteristic absorption bands at approximately 370–380 nm and 250–260 nm. Upon coordination with Zn^2+^, the long-wavelength absorption band showed a red shift (indicated by the arrow), a typical spectral feature of ligand-to-metal charge transfer between metal ions and organic ligands [38]. This red shift arises from the altered electron distribution in the conjugated system of quercetin upon coordination, leading to the formation of a stable coordination structure between Zn^2+^ and quercetin [39]. XPS analysis was subsequently employed to elucidate the chemical composition and elemental valence states of the Zn-Q nanozyme [38]. The survey spectrum showed characteristic peaks of Zn, O, and C (Figure 3c). The C1s spectrum (Figure 3d) was deconvoluted into various carbon species, including C-C, C-H, C-O, and C=O, consistent with the chemical composition of the quercetin molecule [40,41]. In Figure 3e, the peak at approximately 531.2 eV was assigned to the oxygen atoms of the coordinated carbonyl groups (C=O), while the peak at 532.5 eV corresponded to the hydroxyl oxygen atoms in C-O groups. The slight shift in the binding energy of O1s was attributed to a decrease in electron cloud density after the coordination of oxygen atoms to Zn^2+^, reflecting the formation of Zn-O coordination bonds [40]. The high-resolution Zn2p spectrum (Figure 3f) displayed two sets of spin–orbit splitting peaks, consistent with the standard binding energy of Zn^2+^ reported in the literature, indicating that zinc in the nanozyme existed stably in the divalent ionic state [42].

**Figure 4:**
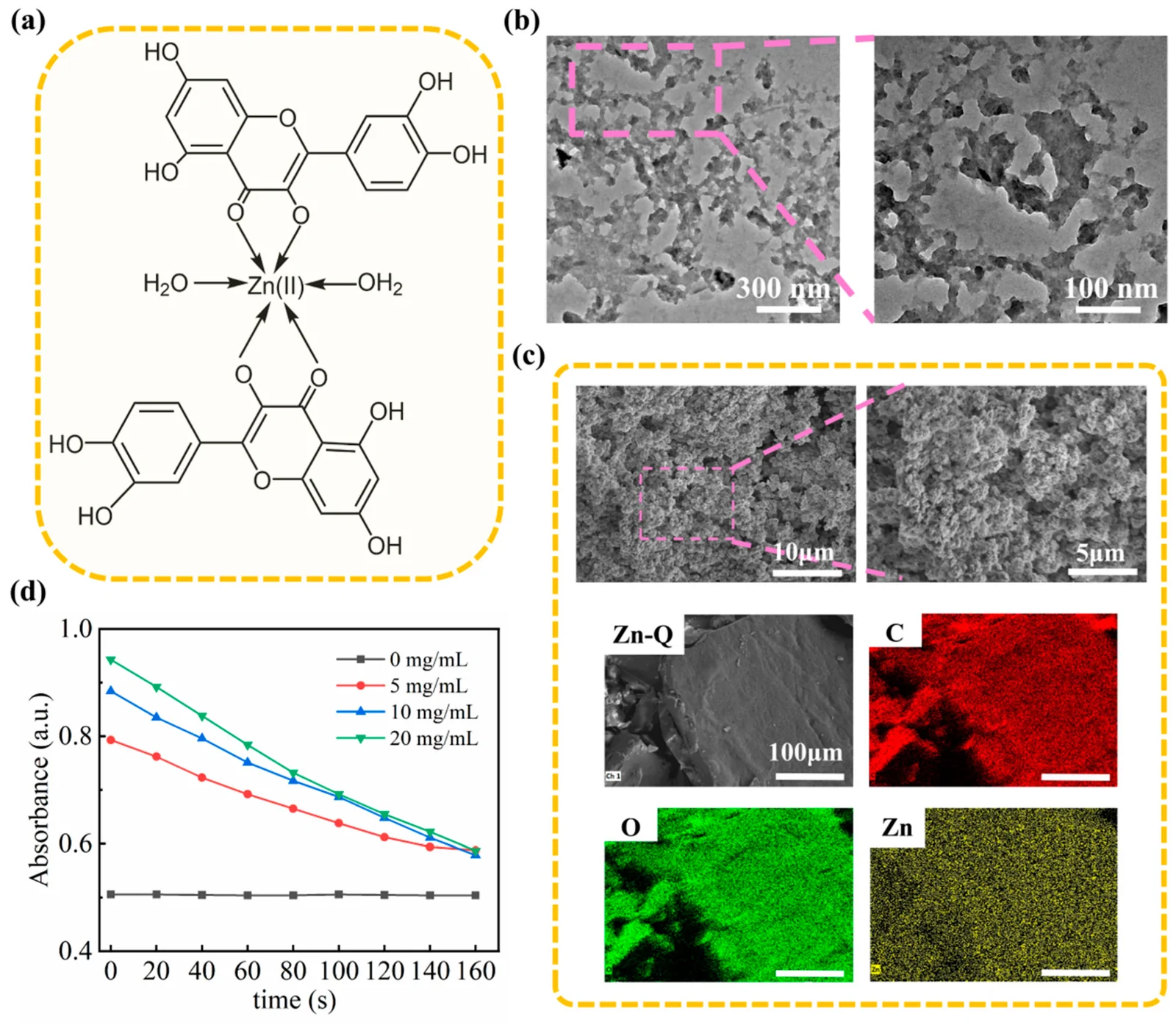
(**a**) Structure of the Zn-Q nanozyme. (**b**) TEM images of the Zn-Q nanozyme. (**c**) SEM image and elemental mapping images of Zn-Q nanozyme. (**d**) CAT-like activity of Zn-Q nanozyme.

The microstructure and elemental distribution of the Zn-Q nanozyme were characterized. As shown in Figure 4a, based on the FT-IR and XPS results described above, the Zn^2+^ ions in Zn-Q coordinated with the carbonyl and hydroxyl oxygen atoms of quercetin, while two water molecules occupied the remaining axial coordination sites, forming a coordinatively saturated octahedral configuration [43]. This coordination mode not only effectively stabilized the valence state of Zn^2+^ but also maximally preserved the conjugated aromatic skeleton of quercetin, thereby providing the essential electron-transfer pathway required for its enzyme-mimetic catalytic function [43]. The TEM images in Figure 4b revealed that the Zn-Q nanozyme exhibited a continuous porous network morphology. This coordination polymer framework consisted of interconnected gray aggregates with an irregular pore-size distribution [44]. Such continuous, irregular porous network aggregates represented a typical morphological characteristic of Zn-Q nanozyme coordination polymers [36]. The porous network structure formed via the metal-polyphenol coordination assembly strategy exposes more active sites and promotes the diffusion and transport of substrate molecules [36]. SEM imaging revealed the porous microstructure of the Zn-Q nanozyme, and EDS mapping confirmed uniform zinc distribution throughout the scanned region without enrichment or segregation (Figure 4c), consistent with XPS analysis. This homogeneous elemental distribution ensures uniform accessibility of active sites, preventing localized substrate depletion. Consequently, this structure–function correlation underpins the incorporation of Zn-Q into the SilMA/HA-SH hydrogel network, ensuring consistent bioactivity throughout the composite [44]. The catalytic activity of the nanozyme serves as the fundamental basis for its incorporation as a functional enhancer into the hydrogel system. Previous studies have demonstrated that metal-ligand systems formed by coordinating flavonoid compounds with transition-metal ions can mimic the catalytic mechanisms of natural metalloenzymes. In Figure 4d, the absorbance of the control group (0 mg/mL) remained nearly constant over 160 s, indicating negligible substrate self-decomposition. Upon the addition of Zn-Q nanozyme, the absorbance decreased significantly over time, and the rate of decrease accelerated markedly with increasing Zn-Q nanozyme concentration, exhibiting typical concentration-dependent enzymatic characteristics. Notably, the initial absorbance of the Zn-Q nanozyme groups was generally higher than that of the control group. This was attributed to the intrinsic coloration of the Zn-Q nanozyme, which deepened the color of the detection solution and thereby increased the initial absorbance readings. These results confirm that the Zn-Q nanozyme possesses CAT-like catalytic activity [45]. The porous network morphology of the Zn-Q nanozyme is intrinsically linked to its catalytic performance. This continuous porous architecture, formed through metal-polyphenol coordination assembly, confers two key advantages for enzyme-mimetic catalysis. First, the porous structure endows the nanozyme with a large specific surface area, exposing abundant active sites, including coordinatively unsaturated Zn^2+^ centers and the conjugated aromatic skeleton of quercetin, both of which are essential for substrate binding and electron transfer during catalysis. Second, the interconnected porous channels facilitate the diffusion and transport of substrate molecules (e.g., H_2_O_2_) to these active sites, thereby minimizing mass-transfer limitations and enhancing overall catalytic efficiency. This characteristic provides a basis for subsequent loading of Zn-Q nanozyme into the SilMA/HA-SH hydrogel network, thereby endowing the composite material with biological functions, including antioxidant and pro-repair activities [46].

### 2.2. Physicochemical Properties of the Composite Hydrogels

**Figure 5:**
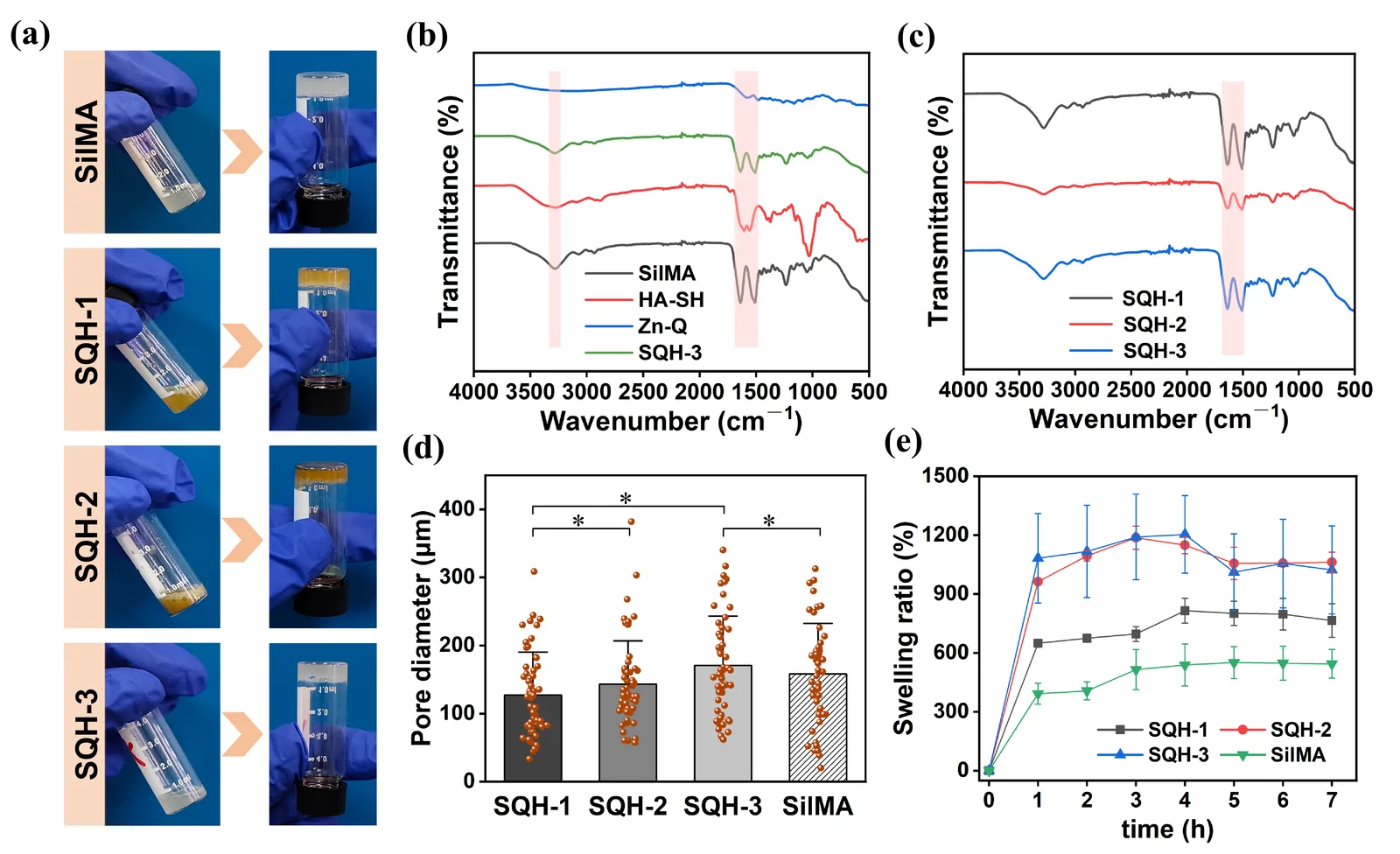
Synthesis and characterization of hydrogels. (**a**) Gelation photographs of SilMA, SQH-1, SQH-2, and SQH-3 hydrogels. (**b**) FT-IR spectra of different components. (**c**) FT-IR spectra of hydrogels with different formulations. (**d**) Pore size distribution. (**e**) Swelling ratios of hydrogels. * *p* < 0.05.

**Figure 6:**
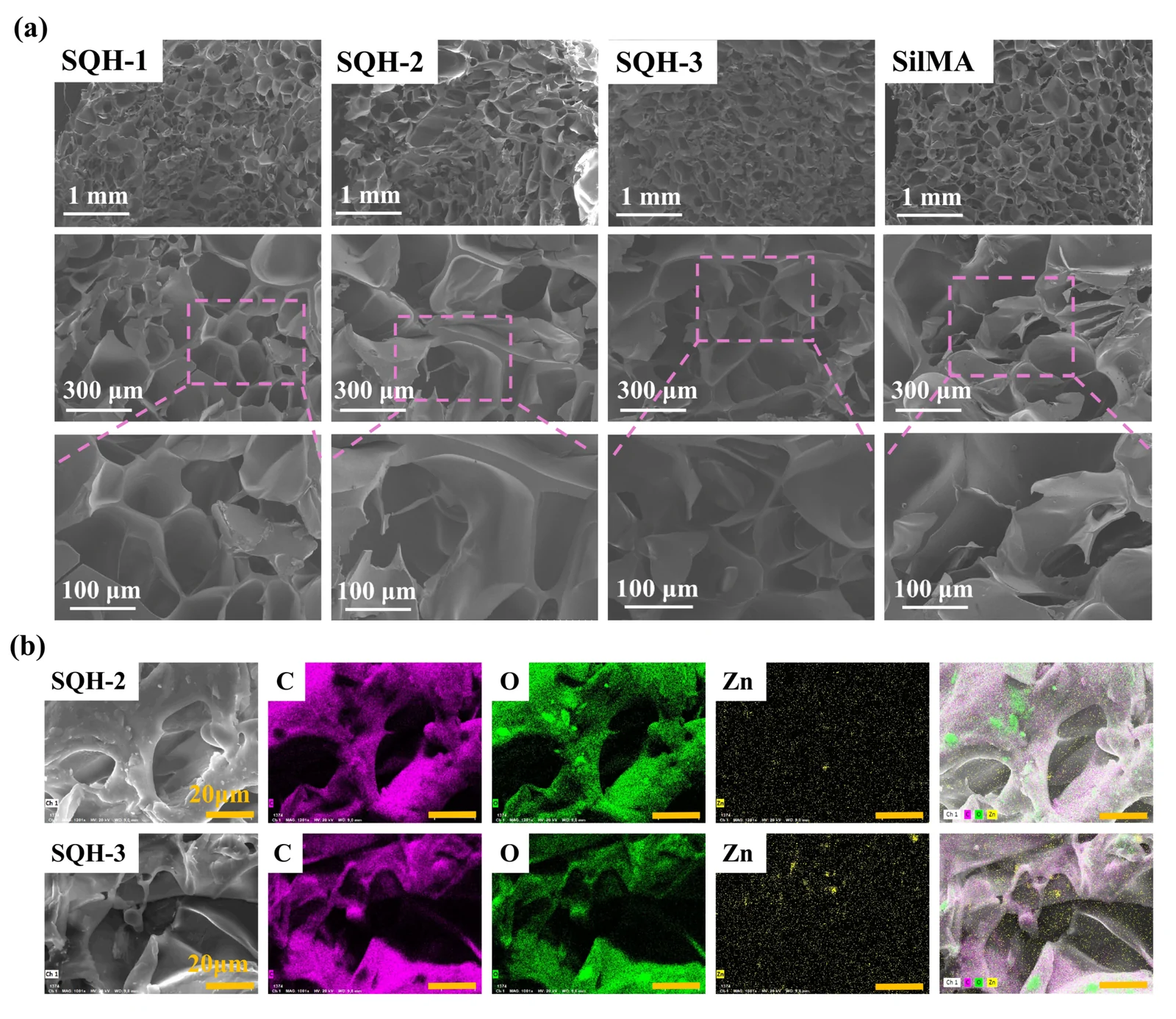
(**a**) SEM images of hydrogels. (**b**) SEM-EDS elemental mapping images of SQH-2 and SQH-3.

As shown in Figure 5a, all hydrogel groups exhibited favorable macroscopic integrity. The FT-IR spectra (Figure 5b) revealed that the composite hydrogels retained the characteristic absorption bands of each component. These included the amide I/II bands of SilMA (1650–1530 cm^−1^), the amide bond peak of HA-SH (1546 cm^−1^), and the phenolic hydroxyl (3261 cm^−1^) and benzene ring skeletal vibration (1500–1600 cm^−1^) signals of Zn-Q nanozyme [47]. No new peaks or significant peak shifts were observed. These findings indicate that all components were successfully integrated through the synergistic action of photocrosslinking and click reactions. The incorporation of Zn-Q nanozyme did not significantly interfere with the chemical crosslinking between SilMA and HA-SH. A further comparison of the FT-IR spectra of SQH-1, SQH-2, and SQH-3 (Figure 5c) showed distinct differences in the 1500–1600 cm^−1^ region (pink shaded area). The absorption intensity of SQH-1 (without Zn-Q nanozyme) was notably weaker. As the Zn-Q nanozyme content increased, the absorption peaks in this region gradually strengthened. Concurrently, the O-H/N-H stretching vibration peak at 3400 cm^−1^ exhibited slight broadening and a red shift. These changes may be attributed to hydrogen bonding and coordination interactions between the phenolic hydroxyl and coordinated carbonyl groups of the Zn-Q nanozyme and the polymer matrix (amide groups of SilMA and carboxyl/hydroxyl groups of HA-SH) [48]. It should be noted that the Zn-Q nanozyme is physically encapsulated within the hydrogel network rather than covalently grafted to the polymer chains. The nanozyme interacts with the SilMA/HA-SH matrix through multiple noncovalent interactions that serve as dynamic and reversible crosslinks. Metal-polyphenol nanocomposites have been reported to enhance the structural stability of hydrogel networks through multiple noncovalent interactions [48]. This physical encapsulation strategy, combined with the dynamic nature of these noncovalent interactions, is particularly advantageous for energy dissipation through reversible bond breakage and reformation under mechanical loading. While the encapsulation efficiency was not quantitatively determined in this study, the Zn-Q nanozyme was incorporated by directly dispersing a known amount of pre-synthesized powder into the precursor solution prior to gelation, such that the hydrogel network forms in situ around the dispersed nanozyme. The homogeneous distribution of Zn-Q confirmed by SEM-EDS mapping (Figure 6b) supports the effectiveness of this physical entrapment strategy. Quantitative determination of encapsulation efficiency and release kinetics will be addressed in future studies.

The pore size distributions of the three hydrogel groups were statistically analyzed based on SEM images (Figure 5d). All groups displayed highly interconnected three-dimensional porous network structures (Figure 6a). Compared with SQH-1, the Zn-Q-loaded SQH-2 and SQH-3 hydrogels showed slightly enlarged pore sizes. This suggests that the intrinsic three-dimensional porous network structure of the Zn-Q nanozyme hindered the dense packing of SilMA and HA-SH polymer chains during gelation, leading to the formation of pores with a looser structure [49,50]. SEM-EDS mapping revealed a homogeneous distribution of Zn throughout the hydrogel matrix of SQH-2 and SQH-3 (Figure 6b), with no detectable aggregation. This suggests uniform dispersion of the Zn-Q nanozyme within the network, which may contribute to the microscale regulation of crosslinking density and pore architecture. The altered pore structure directly influenced the swelling behavior of the hydrogels (Figure 5e). The pure SilMA hydrogel exhibited the lowest equilibrium swelling ratio (547%). After the introduction of hydrophilic HA-SH, the swelling ratio of SQH-1 increased to 764%. With the addition of Zn-Q nanozyme, the swelling ratios of SQH-2 and SQH-3 reached 1056% and 1082%, respectively. These values were significantly higher than those of the other groups. This enhancement stems from two factors: first, the continuous porous network structure of the Zn-Q nanozyme can store and retain substantial amounts of water through capillary action. Second, quercetin molecules are rich in phenolic hydroxyl groups, which can form hydrogen bonds with water molecules [51,52]. Both factors contributed to the improved water absorption capacity of the composite hydrogels.

### 2.3. Rheological Behavior

**Figure 7:**
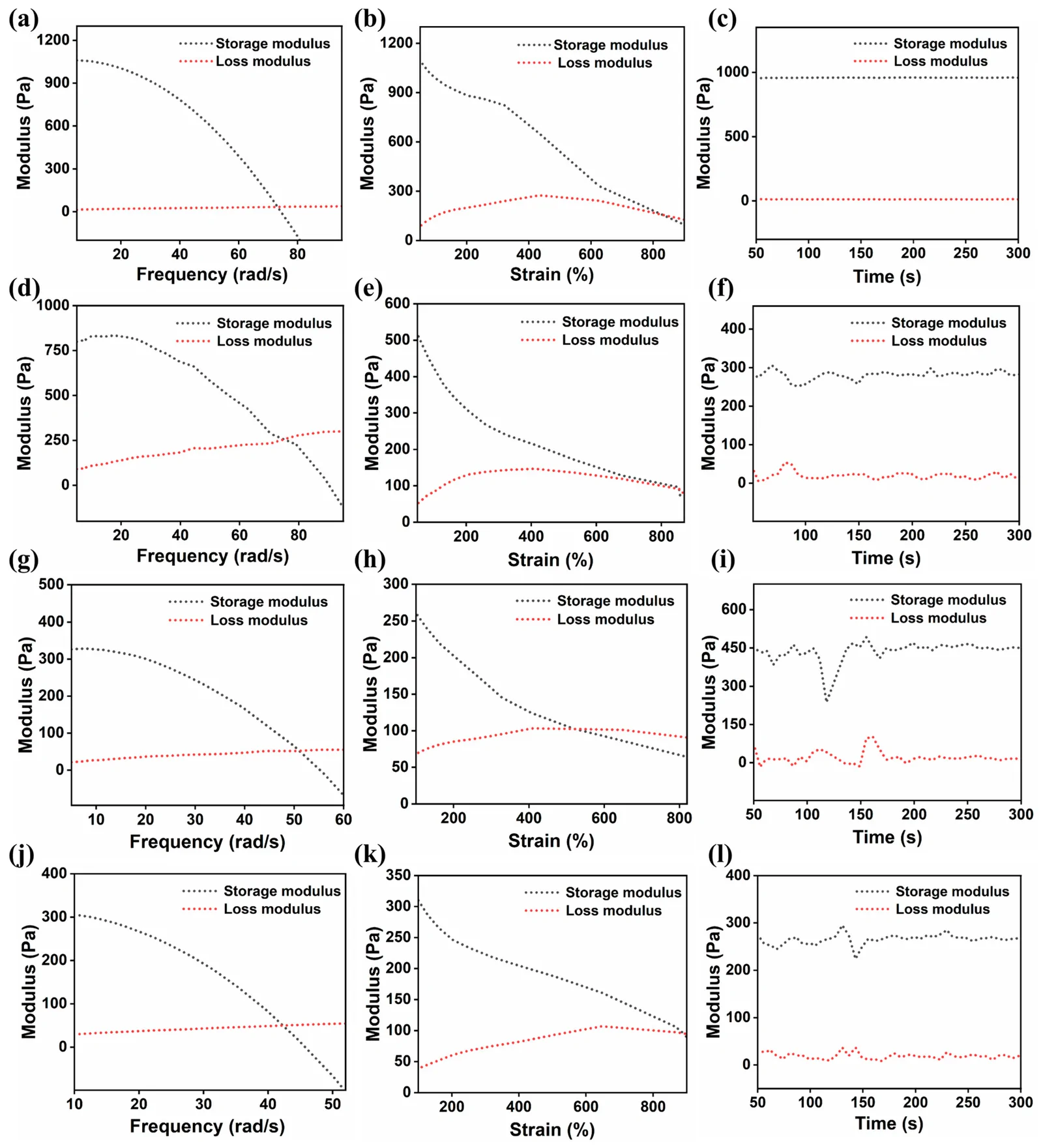
(**a**) Frequency sweep, (**b**) strain sweep, and (**c**) time sweep of SilMA hydrogel. (**d**) Frequency sweep, (**e**) strain sweep, and (**f**) time sweep of SQH-1. (**g**) Frequency sweep, (**h**) strain sweep, and (**i**) time sweep of SQH-2. (**j**) Frequency sweep, (**k**) strain sweep, and (**l**) time sweep of SQH-3.

The rheological properties of SilMA, SQH-1, SQH-2, and SQH-3 hydrogels were characterized by dynamic oscillatory shear measurements. The frequency sweep results (Figure 7a,d,g,j) showed that the storage modulus (G′) was higher than the loss modulus (G″) for all groups. This indicates the successful formation of an elastically dominant three-dimensional crosslinked network. Such a structure can effectively resist energy dissipation under low-frequency perturbations [53]. The pure SilMA hydrogel exhibited the highest G′ (1050 Pa). After the introduction of HA-SH to construct a dual network (SQH-1), G′ decreased to 800 Pa. Upon further loading of the Zn-Q nanozymes (SQH-2 and SQH-3), G′ decreased to 300 Pa. Meanwhile, the G′-G″ crossover points (gel-sol transition frequencies) also exhibited a gradient change. The crossover points for SilMA and SQH-1 were approximately 75 rad/s. For SQH-2 and SQH-3, the crossover points appeared at 52 rad/s and 43 rad/s, respectively. These results suggest that although HA-SH enabled network construction via thiol-ene click reactions, its flexible glycan chains partially diluted the crosslinking density of the SilMA network, thereby increasing the viscous dissipation component of the system. Consequently, the gel transformed from a rigid single network to a dual network with a more balanced viscoelasticity. The incorporation of the Zn-Q nanozyme occupied part of the network space, thereby hindering effective crosslinking between SilMA and HA-SH polymer chains [54]. Notably, the difference between G′ and G″ in the low-frequency region remained relatively stable for SQH-2 and SQH-3. This indicates that hydrogen bonding and coordination interactions between the phenolic hydroxyl and the coordinated carbonyl groups of the nanozyme and the polymer matrix partially compensated for the loss of chemical crosslinking. Thus, the basic integrity of the network was maintained. The strain sweep results (Figure 7b,e,h,k) showed that G′ began to decrease with increasing strain. Concurrently, G″ gradually increased and reached a peak. This indicates that the material entered the nonlinear viscoelastic region. At this stage, the network began to undergo irreversible structural rearrangement or partial cleavage of chemical bonds [55,56]. The time sweep results (Figure 7c,f,i,l) showed that G′ remained higher than G″ throughout the 300 s monitoring period at a constant frequency. All hydrogels maintained favorable gel stability. The decrease in storage modulus upon Zn-Q incorporation reflects a reduction in chemical crosslinking density, as the nanozyme occupies space within the network and partially hinders the efficient crosslinking between SilMA and HA-SH chains. However, the maintained network integrity, as evidenced by the stable G′-G″ difference in the low-frequency region, suggests that noncovalent interactions (hydrogen bonds and metal-coordination bonds) introduced by the Zn-Q nanozyme partially compensate for the loss of chemical crosslinks. This compensation mechanism is expected to play a critical role in the mechanical performance under large deformation.

### 2.4. Mechanical Properties

**Figure 8:**
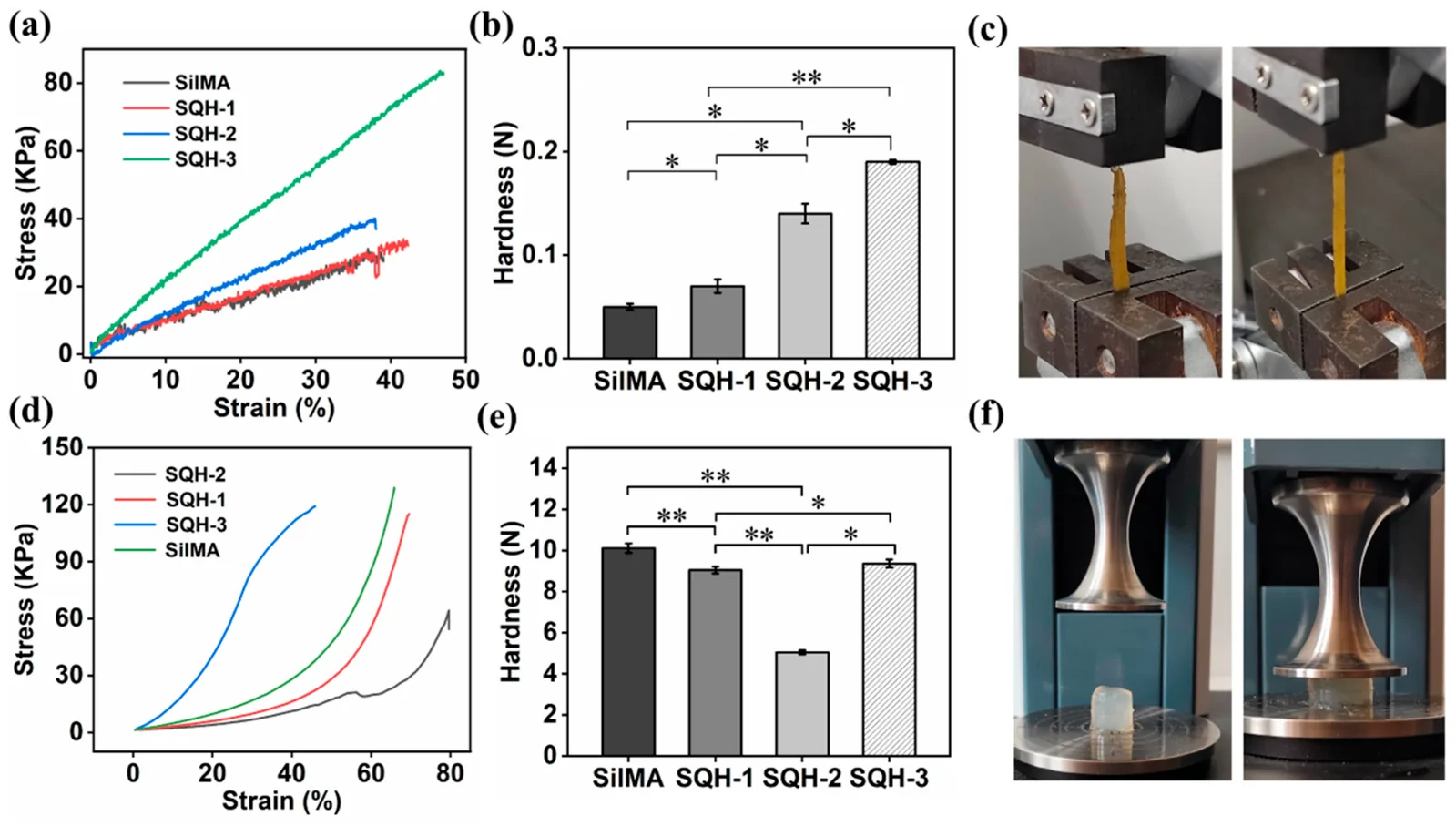
(**a**) Tensile stress–strain curves and (**b**) maximum tensile force of hydrogels. (**c**) Photographs of the hydrogels during tensile testing. (**d**) Compressive stress–strain curves and (**e**) maximum compressive stress of hydrogels. (**f**) Photographs of the hydrogels during compression testing. * *p* < 0.05 and ** *p* < 0.01.

The mechanical properties of hydrogels in relation to target tissues are a critical consideration for evaluating their potential in biological applications. To systematically assess the mechanical properties of the composite hydrogels, tensile (Figure 8a–c) and compression (Figure 8d–f) tests were performed on all groups. As shown in Figure 8a,b, the tensile properties of the SilMA/HA-SH/Zn-Q composite hydrogels displayed a clear composition-dependent behavior. The SilMA hydrogel exhibited low tensile strength, with a maximum tensile force of approximately 0.05 N. This was attributed to the photocrosslinked methacryloyl groups in the SilMA network. The short chain segments between crosslinking points readily generate stress concentration under external force. As a result, the network underwent rapid brittle fracture. After the introduction of the Zn-Q nanozyme, the maximum tensile forces of SQH-2 and SQH-3 increased significantly. The fracture strain of SQH-3 reached 46%. This indicates that incorporating the nanozyme transformed the hydrogel from brittle to tough. This enhancement originated from hydrogen bonding and coordination interactions between the phenolic hydroxyl groups of the Zn-Q nanozyme and the polymer matrix. These noncovalent interactions effectively maintained network extensibility even at a relatively low chemical crosslinking density [57]. The compressive stress–strain curves (Figure 8d) and maximum compressive stress statistics (Figure 8e) further revealed the compressive performance of the hydrogels. SilMA exhibited a compressive stress of approximately 130 kPa at 60% strain, with a maximum force of 10.1 N. Notably, the maximum compressive stress of SQH-2 showed an anomalous decrease, whereas SQH-3 recovered to a higher level. This contrasting mechanical behavior can be attributed to the concentration-dependent reinforcing effect of the Zn-Q nanozyme. As confirmed by SEM-EDS elemental mapping (Figure 6b), the Zn-Q nanozyme was uniformly dispersed within both SQH-2 and SQH-3 hydrogels. However, at the relatively lower loading (100 mg, SQH-2), the nanozyme may not have reached a sufficient concentration to form an integrated reinforcing network, thereby compromising the compressive resistance. In contrast, the higher nanozyme loading in SQH-3 enabled the formation of an effective reinforcing network, thereby restoring and enhancing the hydrogel’s mechanical integrity [58,].

**Figure 9:**
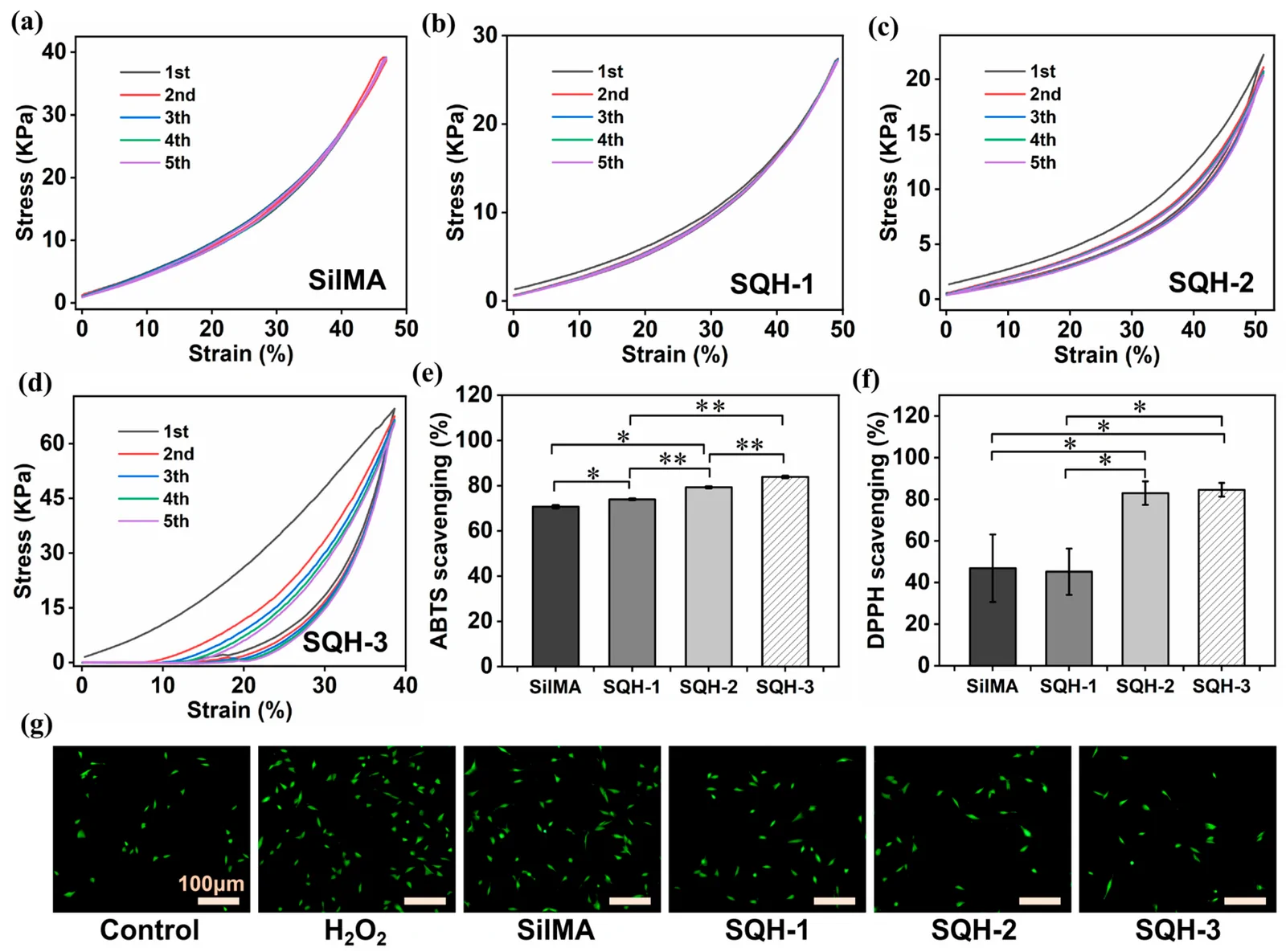
(**a**–**d**) Stress–strain curves of five successive compression cycles. (**e**) ABTS scavenging efficiency and (**f**) DPPH scavenging efficiency of the hydrogels. (**g**) Fluorescence images of DCFH-DA staining after different treatments of NIH-3T3 cells. * *p* < 0.05 and ** *p* < 0.01.

Cyclic compression testing is a key method for evaluating the fatigue resistance and self-recovery capacity of hydrogels. It reveals the energy dissipation characteristics of each group (Figure 9a–d). Five successive cyclic compression tests were performed on all samples. The stress–strain curves of SilMA almost completely overlapped across the five cycles. This hydrogel showed an extremely small hysteresis loop (Figure 9a). These observations indicate excellent elastic recovery and fatigue resistance. The cyclic curves of SQH-1 still maintained good overlap, but the compressive stress decreased slightly (Figure 9b). This is consistent with the rheological results. The introduction of HA-SH chains reduced the crosslinking density to some extent. Incorporation of the Zn-Q nanozyme reduces the effective chemical crosslinking density by sterically occupying network volume and impeding efficient crosslinking between SilMA and HA-SH chains, resulting in a lower G′ [59]. In contrast, SQH-2 and SQH-3 exhibited a pronounced Mullins softening effect under continuous loading [60]. The stress in the first cycle was significantly higher than in subsequent cycles (Figure 9c,d). For SQH-3 in particular, the cyclic curves showed obvious stress decay and an enlarged hysteresis loop. The Zn-Q nanozyme introduces numerous reversible noncovalent interactions, including hydrogen bonds between phenolic hydroxyl groups and polymer chains and metal coordination bonds between Zn^2+^ and the catechol groups of quercetin. Under large deformations (tension or compression), these weak bonds preferentially rupture, dissipating substantial mechanical energy and thereby shielding the covalent network from catastrophic failure. In subsequent cycles, the rupture-recombination of these sacrificial bonds reached a dynamic equilibrium. The stress–strain curves then stabilized. Consequently, despite the reduced storage modulus, the Zn-Q-loaded hydrogels achieve high tensile toughness and energy-dissipation capacity through dynamic noncovalent interactions that act as energy-dissipation units. This imparts favorable fatigue resistance to the material [57]. The high loading of the Zn-Q nanozyme in SQH-3 (200 mg) provided abundant dynamic coordination crosslinking points. This enabled substantial energy dissipation during the initial loading. However, it also caused significant stress softening during the early cycles [61]. Despite the initial stress decay, the residual stress of SQH-3 after five cycles remained higher than that of SQH-2. The curves also tended to stabilize. This indicates that the high nanozyme dosage endowed the network with a stronger energy-dissipation capacity. This “sacrificial bond” mechanism is highly consistent with the fatigue behavior reported for chemically-physically dual-network hydrogels. The SilMA/HA-SH covalent skeleton provides elastic restoring force. The Zn^2+^-quercetin dynamic coordination network serves as the sacrificial component for energy dissipation. The synergistic action of both components enables the hydrogel to withstand large deformations without catastrophic failure [21]. The tensile and compression results demonstrate that the mechanical properties of the composite hydrogels can be regulated over a wide range by adjusting the Zn-Q nanozyme content. This allows the strength, toughness, and fatigue resistance to be tailored to meet the requirements of different tissue repair scenarios.

### 2.5. Antioxidant Properties of Hydrogels

Excessive reactive oxygen species (ROS) disrupt the homeostasis of the tissue microenvironment and inhibit cell proliferation and migration. Therefore, the development of hydrogel materials with both efficient antioxidant capacity and suitable mechanical properties is essential for regulating oxidative stress and promoting tissue regeneration [62]. DPPH and ABTS were employed as two complementary in vitro radical scavenging models. These models quantitatively evaluated the antioxidant efficacy of the hydrogels from lipophilic and hydrophilic perspectives, respectively. As shown in Figure 9e, in the ABTS radical scavenging assay, SilMA and SQH-1 exhibited scavenging rates of approximately 70%. In contrast, Zn-Q-loaded SQH-2 and SQH-3 exhibited scavenging rates of approximately 79.3 ± 0.2% and 83.8 ± 0.3%, respectively. This demonstrates a significant nanozyme dose-dependent enhancement. The DPPH radical scavenging assay (Figure 9f) showed an even more pronounced improvement. The scavenging rates of SilMA and SQH-1 were only approximately 45%. SQH-2 rose sharply to approximately 83 ± 5.7%, and SQH-3 further reached approximately 84.6 ± 3.3%. The consistent trends across both models confirm the reliability of the composite hydrogels’ antioxidant function. The more substantial improvement in the DPPH assay indicates that the Zn-Q nanozyme exhibits a stronger scavenging preference for lipophilic radicals. This may be related to the hydrophobic aromatic skeleton of the quercetin ligand. This structure facilitates penetration of lipid membranes or interaction with hydrophobic radicals. Intracellular reactive oxygen species (ROS) levels were detected using the DCFH-DA fluorescent probe to evaluate the hydrogels’ ROS-scavenging efficiency. As shown in Figure 9g, the control group exhibited only weak green fluorescence. In contrast, the H_2_O_2_-treated group displayed intense green fluorescence, indicating successful establishment of the oxidative stress model. No obvious change in green fluorescence intensity was observed in the SilMA group compared with the H_2_O_2_-treated group. The SQH-1 group showed a slight decrease, whereas the SQH-2 and SQH-3 groups exhibited markedly attenuated fluorescence. These results demonstrate that Zn-Q-loaded SQH hydrogels possess a robust intracellular ROS-scavenging capacity. The antioxidant capacity of the hydrogels originates from the multiple synergistic mechanisms of the Zn-Q nanozyme. Quercetin is a natural flavonoid compound. Its ortho-diphenolic hydroxyl groups can directly scavenge radicals via a single-electron transfer (SET) mechanism. Additionally, the Zn-Q nanozyme exhibits catalase (CAT)-like activity. It can catalyze the decomposition of H_2_O_2_ into H_2_O and O_2_. This catalytic function complements the direct radical scavenging activity of quercetin. Metal-polyphenol coordination nanozymes have been reported to achieve efficient ROS regulation through a synergistic dual-pathway mechanism of “direct scavenging-enzymatic decomposition” [63]. Notably, combined with the mechanical performance analysis presented earlier, SQH-3 achieved optimal antioxidant efficacy while maintaining high tensile toughness and compressive strength. This reflects the synergistic optimization of functional and mechanical properties. Such composite hydrogels, which combine efficient antioxidant capacity with suitable mechanical performance, can simultaneously provide ROS scavenging and mechanical support in oxidative-stress microenvironments (such as diabetic wounds). This provides an integrated solution for tissue repair [64]. However, it is important to note that the CAT-like activity observed in Figure 4d pertains to the free Zn–Q nanozyme. Whether this enzymatic activity is fully preserved within the hydrogel matrix remains to be verified.

### 2.6. Antibacterial Properties of Hydrogels

**Figure 10:**
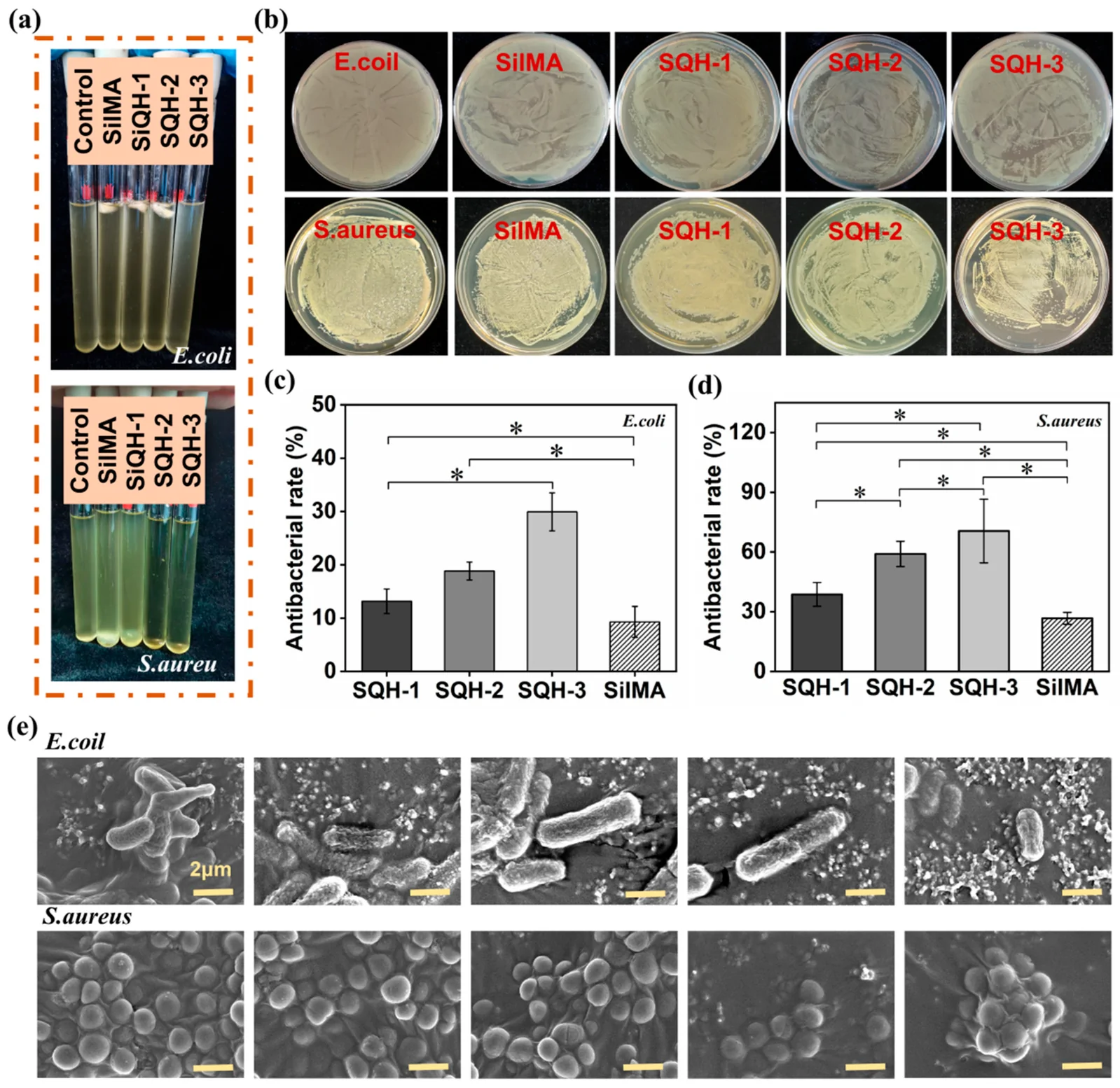
Antibacterial performance of the hydrogels. (**a**) Photographs of bacterial suspension turbidity after 6 h co-culture with hydrogels. (**b**) Photographs of agar plate cultures after 12 h. Antibacterial rates against (**c**) *E. coli* and (**d**) *S. aureus*. (**e**) SEM images of bacteria. * *p* < 0.05.

Infection is one of the leading causes of wound healing failure. Bacterial biofilm formation impedes re-epithelialization and induces chronic inflammation. Therefore, hydrogel materials with effective antibacterial activity are of great significance for wound management [65]. Figure 10a shows changes in liquid turbidity following co-culture of various hydrogels with bacterial suspensions. Compared with the control group, the SilMA group showed almost no change in turbidity. This indicates that the pure SilMA hydrogel itself does not possess significant antibacterial capacity. The SQH-1 group showed a slight decrease in turbidity. In contrast, the bacterial suspensions in the SQH-2 and SQH-3 groups were distinctly clear and transparent. The bacterial suspensions after co-culture were spread on agar plates; colony growth after 12 h (Figure 10b) closely matched the turbidity trends. Dense colonies were still visible on the SilMA and SQH-1 plates; the colony numbers on the SQH-2 and SQH-3 plates were significantly reduced; and the antibacterial effect of SQH-3 was the most prominent. Quantitative statistical analysis (Figure 10c,d) further confirmed these observations. For *E. coli*, the antibacterial rates of SQH-2 and SQH-3 were 18.8 ± 1.7% and 29.9 ± 3.6%, respectively. For *S. aureus*, the antibacterial rates of SQH-2 and SQH-3 were approximately 59.1 ± 6.3% and 70.5 ± 15.3%, respectively. The observed dose-dependent antibacterial activity of Zn-Q-loaded hydrogels against both E. coli and S. aureus is consistent with previous reports on zinc-based SF/HA wound dressings. Hadisi et al. demonstrated that zinc oxide incorporated into SF/HA electrospun nanofibers exhibited a dose-dependent antibacterial effect, with higher zinc content leading to enhanced bacterial inhibition. This consistency suggests that the antibacterial mechanism of zinc-based SF/HA systems may be broadly applicable across different material formats, from electrospun fibers to hydrogel networks [31]. Notably, all hydrogel groups exhibited stronger inhibitory effects against *S. aureus* than against *E. coli*. This difference can be attributed to the essential structural differences between the two bacterial cell walls. Gram-positive bacteria possess a thick but relatively simple peptidoglycan layer; they lack the barrier protection of lipopolysaccharides (LPS) in the outer membrane of Gram-negative bacteria [66]. Consequently, the Zn^2+^ ions and quercetin molecules released from the Zn-Q nanozyme can more readily penetrate and disrupt the cell wall integrity of *S. aureus* [67]. SEM images (Figure 10e) revealed the antibacterial mechanism at the microscopic level. The bacteria in the control group showed intact morphology and smooth surfaces; they displayed typical rod-shaped (*E. coli*) or spherical (*S. aureus*) characteristics [68]. After treatment with SilMA and SQH-1, the bacterial morphology showed no obvious changes. After treatment with SQH-2 and SQH-3, the *E. coli* cells exhibited obvious shrinkage and indentation. The *S. aureus* cells underwent membrane collapse and aggregation [68,69]. These morphological changes indicate that the antibacterial mechanism of the Zn-Q nanozyme involves disruption of the bacterial cell membrane [70]. It is worth noting that SQH-3 exhibits a modest antibacterial efficacy against *E. coli*, which is substantially lower than that of recently reported SF- or HA-based wound dressings, such as the SF/HA/Rhe hydrogel (> 94% against both *E. coli* and *S. aureus*) and the PVA-borax/SF/TA/CS hydrogel (84.3% and 98.1% against *E. coli* and *S. aureus*, respectively) [71,72]. However, the integration of potent antioxidant activity with modest antibacterial properties represents a balanced, multifunctional paradigm for chronic wound management. Excessive antimicrobial pressure is not invariably warranted and may inadvertently compromise the commensal microbiota essential for wound repair [73]. Moreover, complete bacterial eradication is not always the most advantageous therapeutic strategy in chronic wound care. Instead, reducing the bacterial load to a threshold that the host immune system can manage, while simultaneously mitigating oxidative stress, fosters a microenvironment conducive to tissue regeneration. This therapeutic balance positions our hydrogel as particularly advantageous for chronic wounds in which oxidative stress serves as the primary pathological driver.

### 2.7. Adhesion Properties of Hydrogels

**Figure 11:**
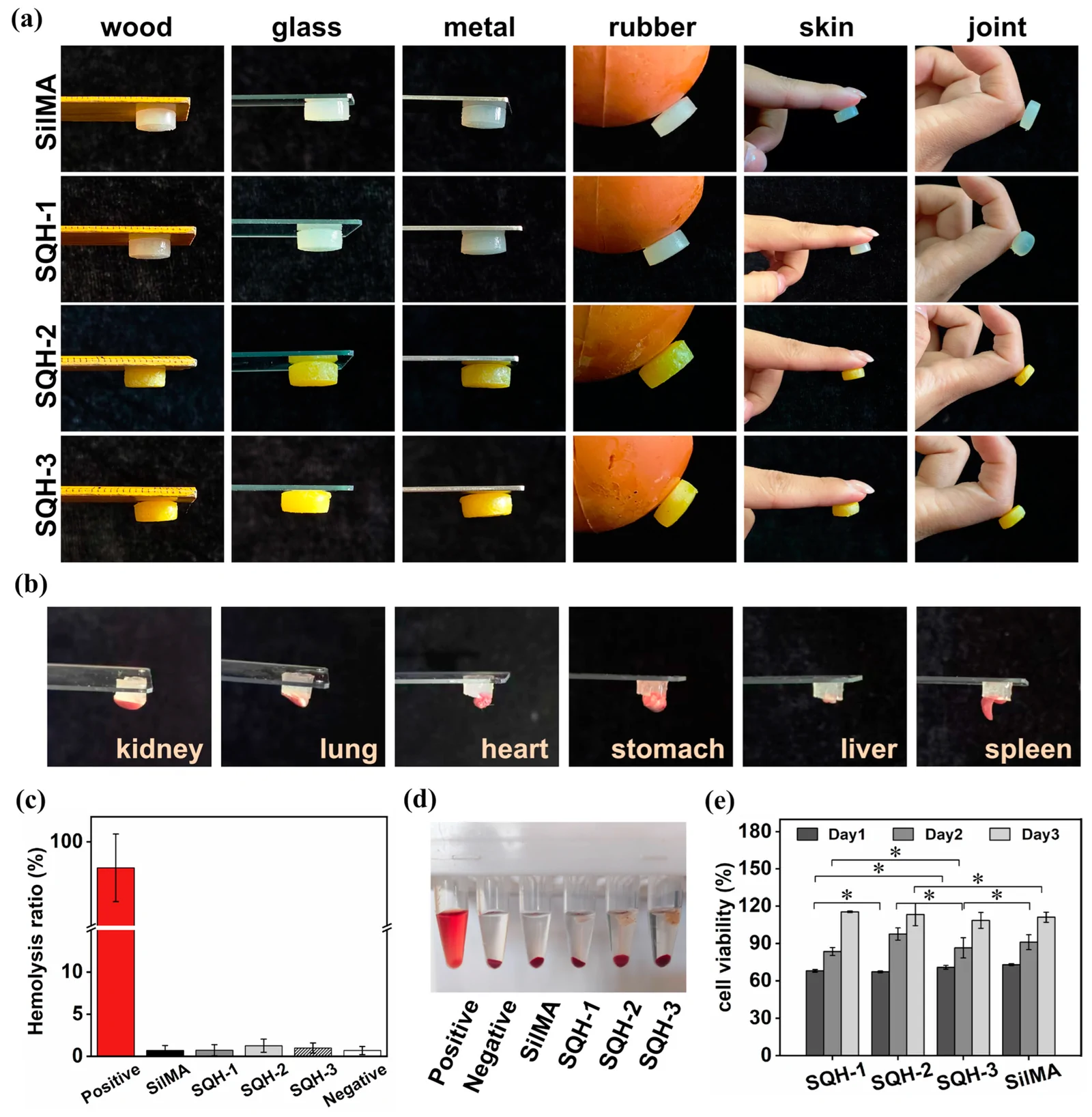
(**a**) Photographs showing adhesion of SilMA, SQH-1, SQH-2, and SQH-3 hydrogels to different materials. (**b**) Photographs of hydrogel adhesion to different organs. (**c**) Hemolysis rates and (**d**) hemolysis photographs of the hydrogels. (**e**) Cell viability of NIH-3T3 after co-culture with hydrogel extracts for 1, 2, and 3 days, as determined by the CCK-8 assay. * *p* < 0.05.

Good tissue adhesion is a key functional property for hydrogels. It enables stable retention in wet physiological environments, effective wound coverage, and promotion of tissue repair. The adhesion performance of the composite hydrogels was evaluated. The hydrogels showed excellent adhesion to various organ tissues and different materials. They adhered firmly to the surfaces of wet internal organs, including the heart, liver, spleen, lung, kidney, and stomach (Figure 11b). They are also attached to heterogeneous material interfaces, such as wood, glass, metal, and rubber (Figure 11a). In particular, they exhibited good conformability to skin and joint surfaces. This adhesion capacity originated from the abundant phenolic hydroxyl, carboxyl, and amide groups in the hydrogel network. These functional groups achieved excellent adhesion through a combination of hydrogen bonding, hydrophobic interactions, and electrostatic interactions with tissue-surface proteins [74]. Hydrogels based on polyphenol structures have been reported to achieve universal adhesion to various substrates through such multiple interactions [75].

### 2.8. Biocompatibility of Hydrogels

Biocompatibility is a critical criterion for evaluating whether hydrogel materials can be safely and effectively applied in biomedicine. It directly relates to the safety and functional durability of materials in vivo. The hemolysis assay results are shown in Figure 11c, the hemolysis rates of SQH-1, SQH-2, and SQH-3 were 0.8 ± 0.6%, 1.3 ± 0.7%, and 1 ± 0.6%, respectively (all below the 5% safety threshold for biomaterials). Figure 11d shows that the supernatant of the positive control group was bright red. In contrast, the supernatants of all hydrogel groups and the negative control group remained clear or pale pink. Intact red blood cell precipitates were visible at the bottom of these tubes. These results indicate that the hydrogels caused no significant damage to red blood cells. They exhibited favorable blood compatibility. This favorable blood compatibility arises from two factors. First, both SilMA and HA-SH are natural polymer derivatives with good biological inertness [76]. Second, the Zn^2+^ ions in the Zn-Q nanozyme exist in a stable coordination state, which prevents the burst release of free Zn^2+^ and thus avoids potential membrane damage or oxidative stress in red blood cells [76,77].

**Figure 12:**
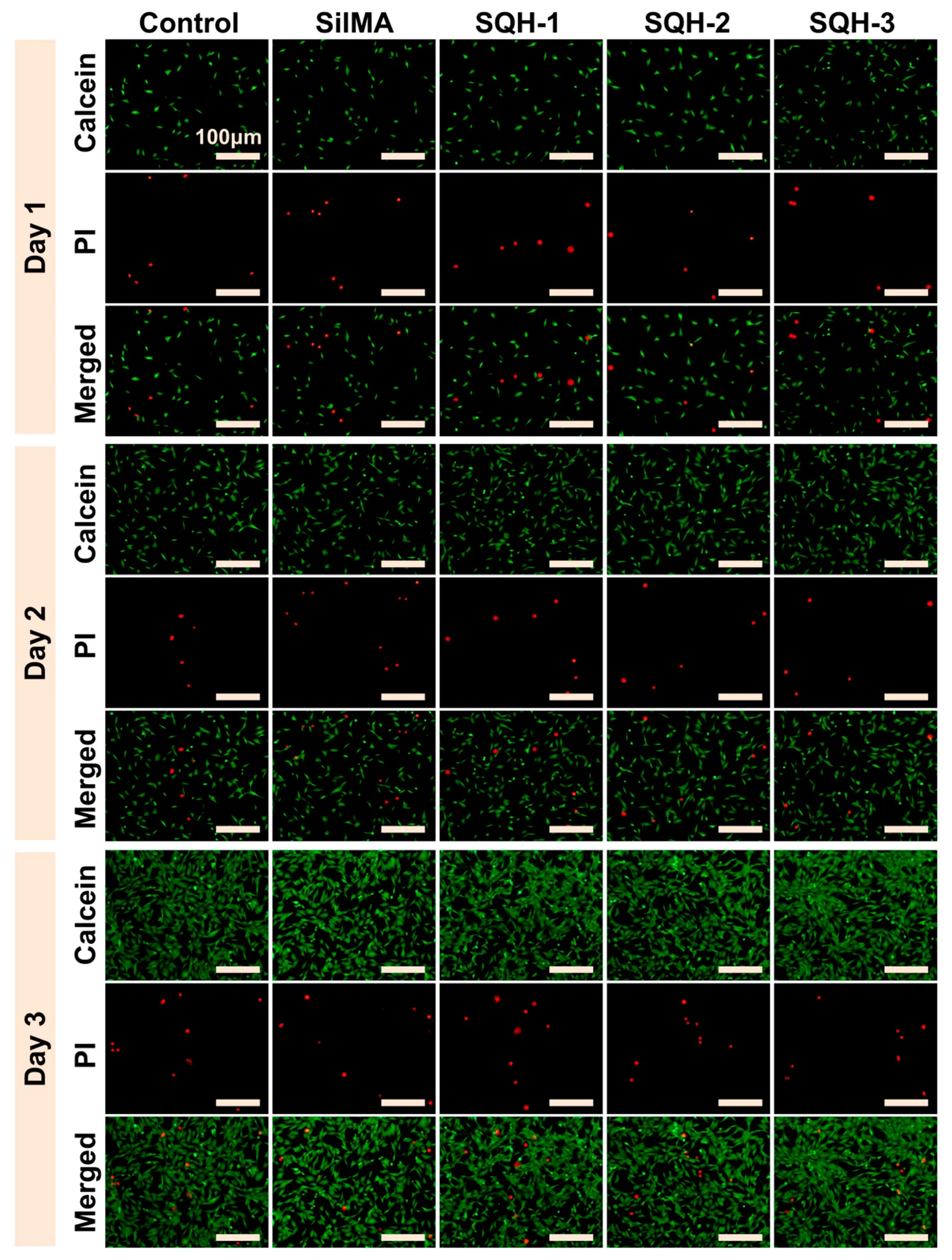
Images of NIH-3T3 cells cultured with hydrogel extract for 1, 2, and 3 days, stained with Live/Dead fluorescence.

The CCK-8 assay was further employed to evaluate the cytotoxicity of hydrogel extracts. This assay measures the effect of hydrogel extracts on cell viability. The results are shown in Figure 11e. At 1 d, the cell viability of all groups remained above 60%. As the culture time increased to 2 d and 3 d, cell viability across all groups increased significantly. By 3 days, the cell viability of all groups exceeded 100%. This indicates that the hydrogel extracts were not only non-cytotoxic but also provided a favorable nutritional microenvironment for cell proliferation. Furthermore, the live/dead cell staining results in Figure 12 validated the above trends. All hydrogel groups showed abundant green fluorescence, indicating viable cells. The green fluorescence intensity gradually increased with prolonged culture time, and by day 3, only a few red fluorescent signals were observed. Compared with the control group, none of the hydrogel groups exhibited massive cell death. Notably, the cell growth status of the SQH-2 and SQH-3 groups was comparable to that of the control group, demonstrating good pro-proliferative capacity. Silk fibroin and hyaluronic acid are natural polymer materials. Their degradation products, such as amino acid oligopeptides and oligosaccharide fragments, can provide nutritional support for cell metabolism. These materials have been widely shown to possess good cytocompatibility [78]. Notably, although SQH-2 and SQH-3 were loaded with the Zn-Q nanozyme, their cell viability did not decrease significantly compared with SQH-1 and pure SilMA. This indicates that the Zn-Q nanozyme at these concentrations has no obvious cytotoxicity. This safety profile may originate from two mechanisms. First, the Zn^2+^ ions are stably bound to the quercetin skeleton through coordination bonds. This prevents the burst release of free Zn^2+^ and the resulting damage to the cell membrane and oxidative stress [79]. Second, the physical encapsulation of the nanozyme within the three-dimensional hydrogel network further slows the release rate of Zn^2+^. This maintains the Zn^2+^ concentration within a safe range that is tolerable to cells [80]. Additionally, the Zn-Q nanozyme scavenges ROS in the culture medium. This alleviates oxidative stress-induced cell damage and indirectly promotes cell growth [81]. This observation is consistent with the antioxidant assay results described earlier. It should be noted that the present study did not quantitatively evaluate the degradation kinetics of the composite hydrogels under physiological or wound-mimicking conditions, which represents a limitation of the current work. Nevertheless, the chemical composition and crosslinking architecture of this hydrogel system provide a sound basis for predicting its degradation behavior. Both SilMA and HA-SH are derived from naturally occurring biodegradable polymers. HA-SH is susceptible to enzymatic cleavage by hyaluronidases, which are abundantly present in wound tissues, whereas the silk fibroin backbone undergoes proteolytic degradation over time. The resulting byproducts, including amino acid oligopeptides and oligosaccharide fragments, are well documented to be non-cytotoxic and even metabolically beneficial to cells, consistent with our cytocompatibility findings. Moreover, the dynamic nature of the noncovalent interactions (hydrogen bonds and Zn^2+^-quercetin coordination bonds) imparts additional responsiveness to the network, potentially enabling controlled degradation modulated by the wound microenvironment. Future work will therefore focus on systematically characterizing the degradation profiles of these hydrogels under various conditions, including enzymatic degradation assays and in vivo degradation monitoring, to fully elucidate their long-term safety and functional durability for wound-healing applications.

## 3. Conclusions

In summary, we have successfully constructed a multifunctional SilMA/HA-SH/Zn-Q composite hydrogel via photocrosslinking and thiol-ene click chemistry. The incorporated Zn-Q nanozyme endows the hydrogel with synergistic antioxidant and antibacterial activities through a dual-pathway mechanism (direct radical scavenging and CAT-mimetic catalysis), while simultaneously modulating mechanical properties via dynamic coordination bonds. The resulting hydrogel exhibits favorable viscoelasticity, tunable mechanics, tissue adhesion, and excellent cytocompatibility. Notably, Zn-Q loading does not compromise but rather promotes cell proliferation through effective ROS scavenging. While these in vitro findings underscore the broad potential of this platform, definitive assessment of clinical utility necessitates comprehensive preclinical validation, including evaluation in standardized animal wound models, degradation kinetics, and in vivo residence time. Successful translation of these results would position the SilMA/HA-SH/Zn-Q hydrogel as a robust candidate for chronic wound management and related regenerative therapies.

## 4. Materials and Methods

### 4.1. Materials

Sodium carbonate anhydrous (Na_2_CO_3_, ≥99.9%), quercetin (97%), sodium hyaluronate (HA, ≥99.9%), methacrylic anhydride (MA), cysteine hydrochloride (98%), potassium persulfate (K_2_S_2_O_8_, 99.5%), and 2,2′-azino-bis(3-ethylbenzothiazoline-6-sulfonic acid) diammonium salt (ABTS) were obtained from Macklin Co., Ltd. (Shanghai, China). Lithium bromide (LiBr, ≥99.9%), N-(3-dimethylaminopropyl)-N′-ethylcarbodiimide hydrochloride (EDC, 98%), and N-hydroxysuccinimide (NHS, ≥98%) were purchased from Aladdin Co., Ltd. (Shanghai, China). Irgacure 2959 was supplied by BASF (Ludwigshafen, Germany). Zinc nitrate hexahydrate (Zn(NO_3_)_2_·6H_2_O, ≥99.9%) was purchased from Xiaoxian Chemical Reagent Factory. Ultrapure water was prepared using a UPT-I-10T Reagent Water System (ULUPURE, Chengdu, China). *Escherichia coli* (*E. coli*, ATCC 25922) and *Staphylococcus aureus* (*S. aureus*, ATCC 6538) were obtained from the American Type Culture Collection (ATCC, Manassas, VA, USA). Sterile anticoagulated sheep blood was purchased from Hunan BKMAM Holding Co., Ltd. (Changsha, China). All reagents were employed in their as-received state without additional purification.

### 4.2. Preparation of Methacrylated Silk Fibroin (SilMA)

Methacrylated silk fibroin (SilMA) was prepared through a sequential process involving degumming, dissolution, graft modification, and purification [82]. To remove sericin, 10 g of sliced silkworm cocoons were boiled in 500 mL of 0.05 M sodium carbonate solution at 100 °C for 30 min. The degummed silk was washed thoroughly with ultrapure water until it turned completely white, and then dried in an oven to obtain degummed silk fibroin. Subsequently, 2 g of dried silk fibroin was solubilized in 20 mL of 9.5 M LiBr at 60 °C for 1 h. GMA (2 mL·g^−1^ based on fibroin mass) was then added, and the system was maintained at 60 °C with 600 rpm agitation for 3 h to complete methacrylation. After reaction completion, the mixture was loaded into a dialysis bag (MWCO: 8000–14,000 Da) and dialyzed against ultrapure water for 3 d, thereby removing unreacted species and byproducts. Finally, the resulting solution was lyophilized to yield methacrylated silk fibroin (SilMA).

### 4.3. Preparation of Mercaptolated Hyaluronic Acid (HA-SH)

To prepare mercaptolated hyaluronic acid (HA-SH), 300 mg of hyaluronic acid (HA) was dissolved in 10 mL of ultrapure water with stirring until completely dissolved [83,84]. The solution pH was first tuned to 5.5 with 0.1 M HCl or 0.1 M NaOH. EDC and NHS were then introduced in equimolar amounts, and the mixture was allowed to stand at room temperature for 30 min to activate the carboxyl groups on HA. Then, under light-protected conditions, 1 mL of freshly prepared 12.7% (*w*/*v*) cysteamine hydrochloride solution was slowly added, and the reaction was allowed to proceed for 12–16 h. The crude mixture was subjected to dialysis (12 kDa MWCO, ultrapure water) to remove unreacted species, then lyophilized, yielding HA-SH as the final product.

### 4.4. Preparation of Zinc-Quercetin Nanoenzyme (Zn-Q)

Based on a previously reported method with modifications, the zinc-quercetin nanozyme was prepared as follows [85]. 0.03 m mol quercetin was dissolved in 20 mL ethanol (anhydrous) and added dropwise to 40 mL Tris-HCl buffer (20 mmol, pH 8). In parallel, 0.3 mmol Zn(NO_3_)_2_·6H_2_O was dissolved in 30 mL of ultrapure water and sonicated for 5 min to ensure homogeneity. The zinc nitrate solution was added dropwise to the above mixed solution at a rate of approximately 1 mL/min under stirring (50 °C, 500 rpm). After the addition, the pH of the mixture was adjusted to 7.5–8.0 using 0.01 M sodium carbonate solution. Stirring was continued at 50 °C for 1.5 h, then the temperature was lowered to ambient, and stirring was maintained for an additional hour. The resulting precipitate was recovered by centrifugation and rinsed three times with a 1:1 (*v*/*v*) ethanol/water mixture. The obtained precipitate was dried at 60 °C for 12 h to yield the zinc-quercetin nanozyme.

### 4.5. Preparation of the Composite Hydrogels

First, 15% (*w*/*v*) silMA solution and 1% (*w*/*v*) HA-SH solution were prepared and mixed uniformly at a volume ratio of 3:1 [86]. Under continuous stirring with mild ultrasonication, the nanozyme was added to the above mixture to achieve homogeneous dispersion. Subsequently, the photoinitiator I2959 was introduced and completely dissolved. Upon exposure to 365 nm UV light for 30 s, the solution was cured to form composite hydrogels, which were designated as SQH-1, SQH-2, and SQH-3, respectively (Table 1). For comparison, SF hydrogel was prepared by adding the photoinitiator I2959 solely to the 15% (*w*/*v*) SilMA solution, followed by curing under 365 nm UV light for 30 s.

### 4.6. Characterization

#### 4.6.1. ^1^H Nuclear Magnetic Resonance (^1^H NMR) Analysis

The structures of the two polymers, SilMA and HA-SH, were characterized by ^1^H NMR spectroscopy [87,88]. SilMA and HA-SH were individually solubilized in D_2_O prior to analysis. The resulting solutions were placed in NMR tubes, maintaining the fill height at approximately 4 cm. Spectra were collected using a JEOL ECZ600R/S3 spectrometer (600 MHz, JEOL, Tokyo, Japan).

#### 4.6.2. Fourier-Transform Infrared Spectroscopy (FT-IR) Analysis

FT-IR analysis was conducted on SilMA, HA-SH, and Zn-Q to identify their chemical structures and characteristic functional groups. Measurements were performed using a PerkinElmer Spectrum 2 spectrometer (Thermo Fisher, Nicolelis5, Waltham, MA, USA) fitted with an ATR accessory [71]. Spectra were collected over the 500–4000 cm^−1^ range at 1 cm^−1^ resolution, with 32 scans co-added for each measurement. The background scan was performed on a clean ATR crystal.

#### 4.6.3. Ultraviolet-Visible Spectroscopy (UV–Vis) Analysis

The characteristic absorption of the Zn-Q nanozyme was characterized using a UV–Vis spectrophotometer (PerkinElmer Lambda 365, Shelton, CT, USA) to infer its coordination structure [37,89]. The Zn-Q nanozyme was dispersed in ultrapure water, and an appropriate volume of the dispersion was placed in a quartz cuvette with a 1 cm optical path length, using ultrapure water as the reference blank. The spectra were recorded over a wavelength range of 200–800 nm.

#### 4.6.4. Scanning Electron Microscope (SEM) Analysis

The morphologies of the freeze-dried SilMA hydrogel, SQH hydrogels (SQH-1, SQH-2, and SQH-3), and Zn-Q nanozyme were observed using a field-emission scanning electron microscope (SEM, Hitachi Regulus 8220, Tokyo, Japan). The samples were first attached to conductive tape and then subjected to platinum sputter coating to ensure adequate conductivity prior to observation. Elemental distribution of the Zn-Q nanozyme was subsequently analyzed by EDS mapping. The pore size analysis was performed on the acquired SEM images using ImageJ software (Version 1.53) [71].

#### 4.6.5. Transmission Electron Microscope (TEM) Analysis

The morphology of the as-prepared Zn-Q nanozyme was characterized by transmission electron microscopy (TEM) [90]. Prior to observation, a small amount of Zn-Q nanozyme was dispersed in ultrapure water. An appropriate volume of the resulting dispersion was dropped onto a copper grid and thoroughly dried. The drop-and-dry procedure was repeated to ensure firm attachment of the sample to the grid, after which TEM observation was performed.

#### 4.6.6. X-Ray Photoelectron Spectrometer (XPS) Analysis

XPS analysis was performed on Zn-Q nanozyme using a Thermo Scientific K-Alpha spectrometer (K-ALPHA 0.5EV, Thermo Fisher, Waltham, MA, USA) to determine the surface elemental composition and chemical states. Survey spectra were acquired with a 1.0 eV step size, while high-resolution narrow scans were recorded at 0.05 eV resolution [89]. The acquired data were processed using Avantage software (version 6.9.0) for peak fitting and data analysis.

#### 4.6.7. Catalase-like Activity Assay

The catalase (CAT)-like activity of Zn-Q nanozyme was measured using UV–vis spectrophotometry [91]. Since H_2_O_2_ exhibits a maximum absorption peak at 240 nm, the consumption of H_2_O_2_ was monitored by measuring the change in absorbance at this wavelength. The reaction mixture (total volume 3.0 mL) consisted of Tris-HCl buffer (50 mmol/L, pH 8.0, containing 10 mmol/L H_2_O_2_) and different concentrations of Zn-Q nanozyme (5, 10, 20 mg/mL). For the negative control, an equivalent volume of buffer lacking Zn-Q nanozyme was added. The absorbance at 240 nm was recorded every 20 s for 3 min using a UV–vis spectrophotometer.

### 4.7. Rheological Properties Analysis

Rheological characterization of the composite hydrogels was carried out on a TA DHR-2 rheometer (TA Instruments, New Castle, DE, USA). The specimens were fabricated into 20 mm-diameter thin discs and tested using a 30 mm low-inertia aluminum parallel-plate configuration [92]. The measurements were performed in three modes:

(1) Time sweep: the storage modulus (G′) and loss modulus (G″) were monitored over time at 37 °C, 1% strain, and a constant frequency of 1 Hz.
(2) Strain sweep: Measurements were performed at 37 °C and 1 Hz, sweeping the strain from 0.1% to 800%.
(3) Frequency sweep: performed at 37 °C and 1% strain, with angular frequency ranging from 0.1 to 100 rad/s.

### 4.8. Swelling Analysis

The freeze-dried hydrogels were first weighed to obtain their initial dry mass (*Wc*). They were subsequently immersed in ultrapure water and permitted to swell at ambient temperature. At designated time points, the specimens were retrieved, lightly dabbed with filter paper to eliminate superficial water, and promptly reweighed (*Ws*). This process was repeated until the sample mass no longer increased significantly. Three parallel samples were prepared for each group. The swelling ratio (*SR*) was calculated using the following Formula (1):

(1) $$\mathrm{SR}\;(\%)\;=\frac{W_{s}\;-\;W_{c}}{W_{c}}\;\times \;100\%$$

### 4.9. Tensile Property Testing

The tensile properties of the hydrogels were characterized using a universal testing machine (CMT-4103, SUST Instrument Co., Ltd., Zhuhai, China) [53]. The hydrogel samples were cut into dumbbell-shaped or strip specimens with dimensions of 50 mm (length) × 3.5 mm (width) × 1.5 mm (thickness). Uniaxial tensile tests were performed at a constant crosshead speed of 10 mm/min until sample fracture, and each sample was tested in at least triplicate.

### 4.10. Compressive Property Test

Compression tests were performed using a biological dynamometer (EFL-MT5000, EFL-Tech Co., Ltd., Suzhou, China) equipped with a 100 N load cell [93]. Cylindrical hydrogel specimens (1 cm diameter) were fabricated for mechanical testing. Compression was applied at a constant crosshead speed of 10 mm/min at ambient temperature until failure. Each sample was tested three times. Furthermore, cyclic compression tests were carried out under displacement control. The fixed displacement amplitude was determined based on the results of the single compression tests, and five consecutive cycles were applied.

### 4.11. Antioxidant Activities Analysis

The antioxidant activity of the SQH hydrogels was evaluated by measuring their scavenging abilities toward ABTS and DPPH radicals [94]. ABTS radical scavenging assay: ABTS (7 mM) was combined with an equimolar volume of potassium persulfate (2.45 mM), and the resulting mixture was incubated in the dark at ambient temperature for 12 h. The resulting mixture was then diluted 25-fold with ultrapure water to obtain the ABTS working solution. A 5 mL aliquot of the working solution was added to 30 mg of freeze-dried hydrogel sample and incubated in the dark at 25 °C with shaking at 150 rpm for 30 min. Following incubation, the supernatant was harvested, and its absorbance at 734 nm was recorded on a PerkinElmer Lambda 950 UV–Vis spectrophotometer. DPPH radical scavenging assay: A 0.1 mM DPPH solution was prepared in absolute ethanol. A 3 mL portion of this solution was dispensed onto 5 mg of lyophilized hydrogel and incubated under identical conditions to those employed in the ABTS assay. Following centrifugation, the supernatant was harvested, and its absorbance at 517 nm was recorded. For both assays, the control consisted of the respective working solution without sample addition, and each experiment was performed in triplicate. The radical scavenging rate was calculated according to Equation (2):

(2) $$\mathrm{ABTS}\;(\mathrm{DPPH})\;\mathrm{Scavenging}\;\mathrm{rate}\;(\%)\;=\;\frac{A_{0}-A_{1}}{A_{0}}\;\times \;100\%$$

where *A*_0_ denotes the absorbance of the control group, and *A*_1_ represents the sample group.

### 4.12. Intracellular ROS Scavenging Activity

Intracellular ROS generation was monitored by employing the DCFH-DA fluorescent probe [87]. Briefly, Cells were plated into 6-well plates and allowed to grow for 24 h. The experimental design comprised a negative control, a positive control, and sample treatment groups. All groups except the negative control were exposed to H_2_O_2_ at a final concentration of 1 mM, while the sample group was additionally treated with 10 µL of the hydrogel extract. The cells were first incubated in the dark at 37 °C for 2 h, followed by three PBS washes. Next, 500 µL of DCFH-DA (10 µM) was introduced into each well, after which the cells were subjected to a second dark incubation at 37 °C for 20 min. Finally, the intracellular fluorescence intensity was observed, and representative images were captured using a fluorescence microscope.

### 4.13. Antibacterial Activity Evaluation

The hydrogels were screened for antibacterial efficacy against the Gram-positive bacterium *Staphylococcus aureus* (*S. aureus*, ATCC 6538) and the Gram-negative bacterium *Escherichia coli* (*E. coli*, ATCC 25922) [55]. The procedure was as follows: frozen stock strains were revived, purified, and expanded in culture, after which the bacterial suspensions were adjusted to approximately 2 × 10^6^ CFU/mL with sterile 0.9% saline. To initiate the antibacterial assay, 15 mg of hydrogel was placed in a glass tube with 10 mL LB broth and 10 mL bacterial suspension, then maintained in a shaking incubator at 37 °C for 6 h. Following incubation, aliquots from both the control and sample groups were taken, and their optical density at 600 nm was measured using a UV–Vis spectrophotometer. Subsequently, the bacterial suspensions from the control and sample groups were spread evenly onto nutrient agar plates and cultured at 37 °C in a biochemical incubator for 12 h. The antibacterial performance was further assessed by observing the colony growth on the plates. The antibacterial rate was calculated according to Equation (3):

(3) $$\mathrm{Antibacterial}\;\mathrm{rate}\;(\%)\;=\;\frac{W_{0}\;-\;W_{s}}{W_{0}}\;\times \;100\%$$

where *W*_0_ and *Ws* represent the absorbance values of the control group and the sample group, respectively.

### 4.14. Adhesive Properties Analysis

The adhesion properties of the hydrogels to various substrates were evaluated using a 90° peel test. The selected substrates included skin, glass, wood, natural rubber, and stainless steel sheet, all of which were subjected to a standardized cleaning procedure prior to testing. Hydrogel specimens were prepared to specified dimensions and adhered to each substrate. For dynamic adhesion assessment, the hydrogel was directly attached to finger joints, and the adhesion state during joint movement was recorded photographically. In addition, excised heart, lung, spleen, and kidney tissues were used as substrates to evaluate the wet tissue adhesion capability of the hydrogel.

### 4.15. Hemolysis Test

The in vitro hemolytic performance of the hydrogel was evaluated using the direct contact method to systematically assess its blood compatibility [87]. The experiment was conducted in 1.5 mL centrifuge tubes, with sample, positive control, and negative control groups. For the experimental group, 2.5 mg of hydrogel was dispersed in 1 mL of normal saline (0.9% NaCl). The negative control was treated with 1 mL of normal saline, whereas the positive control received 1 mL of ultrapure water. All samples were then maintained at 37 °C for 30 min. At least three parallel replicates were prepared for each group. Subsequently, 20 μL of sterile anticoagulated sheep blood was added to each tube, mixed gently, and incubated at 37 °C for an additional 1 h. The tubes were subjected to centrifugation at 3000 rpm for 5 min, after which the supernatant was carefully harvested. Absorbance of the collected supernatant was then recorded at 545 nm on a UV–Vis spectrophotometer. The hemolysis rate (*HR*) was calculated using the following Formula (4):

(4) $$\mathrm{HR}\;(\%)\;=\;\frac{H_{s}\;-\;H_{n}}{H_{p}\;-\;H_{n}}\;\times \;100\%$$

where *Hs*, *Hp*, and *Hn* denote the 545 nm absorbance values of the sample group, positive control group, and negative control group, respectively.

### 4.16. Cytocompatibility Test

Cell viability was quantified by the Cell Counting Kit-8 (CCK-8) assay to assess the cytotoxicity of the hydrogels [87]. For the assay, mouse embryonic fibroblasts (NIH-3T3) were seeded into 96-well plates at a density of 5 × 10^4^ cells/mL. Sample wells were supplemented with 100 μL of hydrogel extract, and control wells with 100 μL of complete medium. The plates were then cultured under humidified conditions (37 °C, 5% CO_2_) for 1, 2, and 3 d. At scheduled intervals, 10 μL of CCK-8 solution was dispensed into each well, and the plates were incubated for an additional 2 h. The absorbance was measured at 450 nm using a microplate reader (SpectraMax 190, Molecular Devices). Cell viability was calculated using the following Formula (5):

(5) $$Cell\;viability\;(\%)\;=\;\frac{A_{s}\;-\;A_{0}}{A_{c}\;-A_{0}}\;\times \;100\%$$

where *As*, *Ac*, and *A*_0_ represent the absorbance values of the sample, the control group, and the blank group, respectively.

In addition, cell proliferation was observed using a live/dead staining assay [87]. NIH-3T3 cells were seeded into 6-well plates at a density of 6 × 10^5^ cells/mL. For the sample groups, 500 μL of hydrogel extract was applied; for the control groups, 500 μL of complete medium was used instead. The cells were then maintained under the same culture conditions for 1, 2, and 3 d. Upon completion of incubation, the cells were subjected to fluorescent labeling using a Calcein-AM/PI Live/Dead Cell Staining Kit, followed by image acquisition via an inverted fluorescence microscope.

### 4.17. Statistical Analysis

All measurements were carried out in three independent replicates, with data expressed as the mean ± SD. Statistical comparisons among groups were performed by one-way ANOVA using SPSS (Version 26.0, SPSS Inc., Chicago, IL, USA). A significance threshold of *p* < 0.05 was adopted.

## Figures and Tables

**Table 1:** Composition of SQH hydrogels at different proportions.

Sample	SilMA ^a^	HA-SH ^b^	I2959	Zn-Q
SQH-1	3 mL	1 mL	2%	
SQH-2	3 mL	1 mL	2%	100 mg
SQH-3	3 mL	1 mL	2%	200 mg

^a^ SilMA solution: 15 wt%. ^b^ HA-SH solution: 1 wt%.

## Data Availability

Data will be made available on request.
